# Phosphazene-catalyzed desymmetrization of cyclohexadienones by dithiane addition

**DOI:** 10.3762/bjoc.13.75

**Published:** 2017-04-24

**Authors:** Matthew A Horwitz, Elisabetta Massolo, Jeffrey S Johnson

**Affiliations:** 1 Department of Chemistry, University of North Carolina at Chapel Hill, Chapel Hill, NC 27599-3290, USA

**Keywords:** conjugate addition, cyclohexadienones, dearomatization, desymmetrization, dithiane, iminophosphoranes, organocatalysis, phosphazenes

## Abstract

We report a desymmetrization of cyclohexadienones by intramolecular conjugate addition of a tethered dithiane nucleophile. Mild reaction conditions allow the formation of diversely functionalized fused bicyclic lactones. The products participate in facially selective additions from the convex surface, leading to allylic alcohol derivatives.

## Findings

Desymmetrization has become a well-developed strategy for the construction of complex molecular frameworks [1–6]. Cyclohexadienones are multipurpose synthetic building blocks that have found a central role in desymmetrization methodologies. The functional groups present in these symmetrical molecules allow for a wide array of downstream transformations and they are all formed through a single reaction from cheap and readily available aromatic feedstocks [7–11]. These substrates have been successfully employed in a number of stereoselective desymmetrization reaction manifolds. Intramolecular Michael additions via enamine intermediates have been studied by the Gaunt [12] and Johnson groups [13]. The You group has disclosed methods for the intramolecular addition of amine [14] and bisphenylsulfonyl [15] nucleophiles using bifunctional cinchona alkaloid catalysts. The Sasai and Enders groups used a phosphinothiourea to enable a Rauhut–Currier reaction to form bicyclic enones [16]. The Tian and Lin group used alkyne-tethered cyclohexadienones in an arylrhodation/conjugate addition sequence that enantioselectively delivered oxabicyclo[4.3.0]nonanes [17]; the Lautens and Lan groups have also contributed to the further development of this reaction [18–19]. The Rovis group employed cyclohexadienone hydroperoxides in a chiral phosphoric acid-catalyzed [1,2]/[1,4]-addition cascade [20]. The same group also developed an acyl anion addition promoted by *N*-heterocyclic carbenes (NHC) that furnished bicyclic furanones via Stetter addition [21]; later, the You group developed an extension of this theme using the same catalytic manifold [22]. More recently, the Corey group has enabled the enantioselective conjugate reduction of prochiral cyclohexadienones using copper hydride generated in situ [23]. Inspired by these advances, we sought to develop an alternative and complementary method invoking the dithiane moiety as an established and easily accessible glyoxylate anion surrogate [24–29]. This would in principle provide access to highly functionalized products with orthogonally protected carbonyl groups in a novel glycolic acid scaffold.

**Scheme 1:**
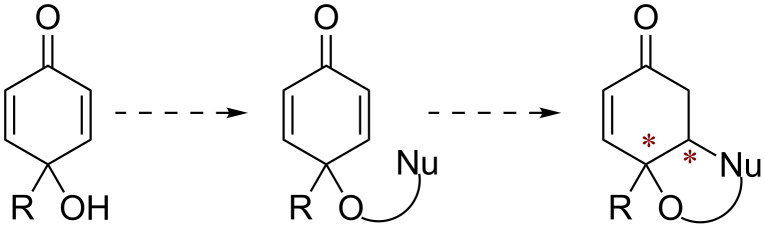
Desymmetrization of cyclohexadienone by tethered nucleophile.

We envisioned utilizing *para*-quinol derivatives featuring a tethered nucleophile as desymmetrization substrates, with the intention of implementing a Brønsted base organocatalyzed addition (Scheme 1). This reaction would lead to bicyclic systems with the salient attribute of having a convex-concave facial differentiation, allowing subsequent diastereoselective transformations. With the aim of using a dithiane nucleophile, we selected 1,3-dithiane-2-carboxylic acid because of its relatively low p*K*_a_ (compared with non-carboxylate substituted analogs) and the possibility of using an ester linkage as a tether. We found that the heretofore unknown dicyclohexylcarbodiimide (DCC) mediated coupling between *para*-quinols and 1,3-dithiane-2-carboxylic acid proceeds in a straightforward manner in cases where R is unbranched (though it does work for R = Ph). Using this method, we were able to easily generate diversely functionalized dithiane-linked *para*-quinols to study the intramolecular cyclization.

**Scheme 2:**
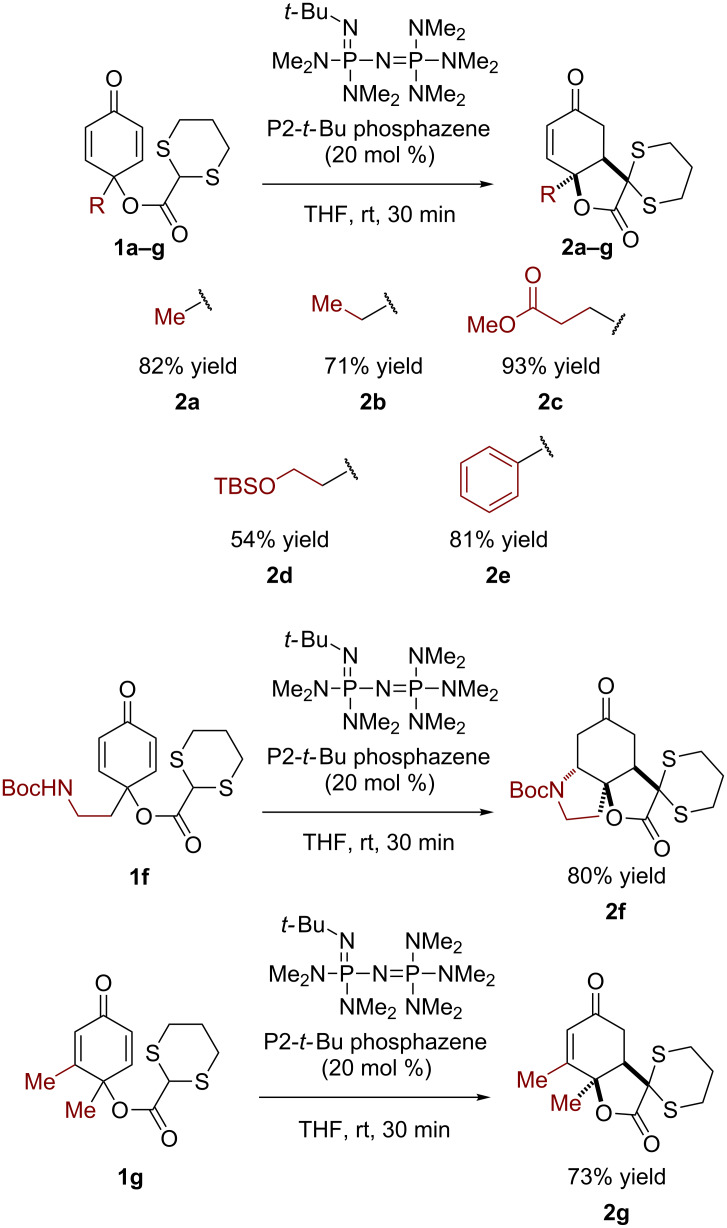
Scope of the transformation.

Based on a prior report [30] demonstrating the efficacy of phosphazene bases in deprotonating carboxylate dithianes, we selected the commercially available achiral superbase P2-*t*-Bu phosphazene to initiate the ring closure (Scheme 2) [31–32]. We found that in the simplest case, with the methyl-substituted *para*-quinol ester (**1a**), the reaction was complete in 30 min at ambient temperature with 20 mol % catalyst [33]. Extending the length of the alkyl chain, the reaction proceeded similarly, even in the presence of a methyl ester or a TBS-protected primary alcohol (**1b–d**); a comparable result was observed with a phenyl substituent (**1e**). We considered that if a nucleophilic group were appended to the *para*-quinol, it would be possible to construct a 5–6–5 fused ring system. Indeed, when R = CH_2_CH_2_NHBoc (**1f**), the desired tricyclic product **2f** was obtained. In all cases only a single diastereomer was observed. In a substrate where a β-methyl group is present on the cyclohexadienone (**1g**), the reaction proved to be completely regioselective, only allowing conjugate addition to the less substituted position.

**Figure 1:**
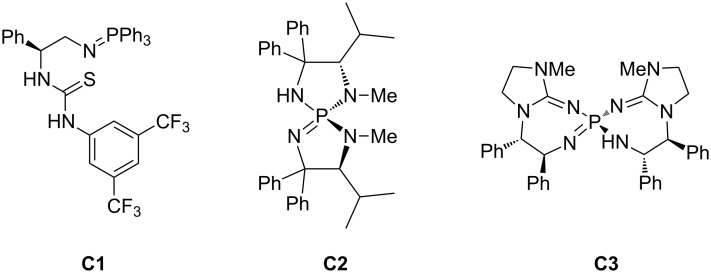
Chiral iminophosphorane catalysts surveyed.

We attempted to render the reaction enantioselective using chiral iminophosphoranes (Figure 1) structurally related to P2-*t*-Bu phosphazene, which are known to be substantially more basic than trialkylamines [34]. With **C1** [34–38] and **C2** [39–55], we observed no product formation, presumably due to insufficient basicity. Though **C3** [30,56–57] led to partial conversion of starting material, no appreciable enantioselectivity was observed.

**Scheme 3:**
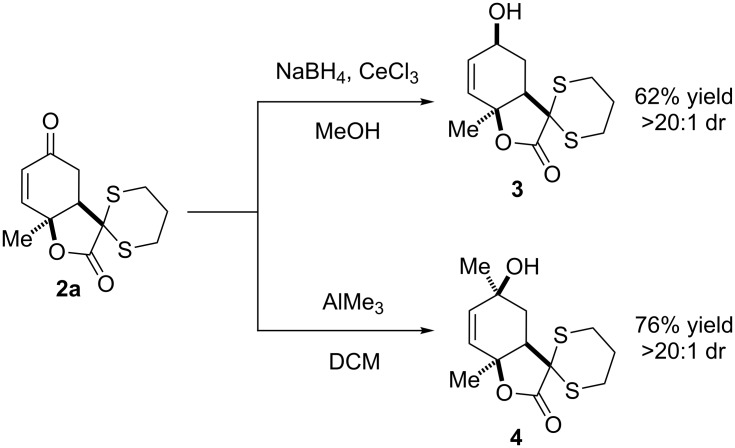
Convex facial additions.

To investigate the feasibility of a convex-facial addition, we subjected **2a** to Luche reduction conditions (Scheme 3). We found this transformation to be completely diastereoselective, and an X-ray diffraction study [58] of the product confirmed our hypothesis regarding the facial selectivity, as the hydride was delivered to the convex face. An analogous reaction occurs when **2a** is treated with AlMe_3_, affording the 1,2-addition product (Scheme 3) [59–63].

We next sought to establish the glyoxylate anion equivalency of the dithiane substructure in our system. In order to reveal the masked carbonyl functionality, we rigorously applied reported dithiane deprotection conditions to **2a** (Table 1). Despite extensive investigations, none of our efforts were fruitful, resulting in either no conversion, side reactions [64], or decomposition. We rationalized these disappointing results considering: 1) the crowded steric environment surrounding the dithiane moiety on the concave face of the bicycle, 2) the sensitive nature of this class of compounds, which stems from the highly reactive functional groups present, and 3) the strained character of the five-membered α-ketolactone (**5**) that would result from deprotection.

**Table 1:** Carbonyl deprotection conditions.

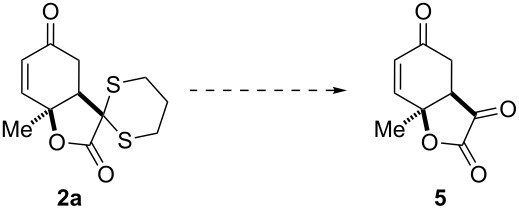		

Entry	Conditions	Result

1 [65]	NBS, MeCN/H_2_O, 0 °C, 10 min^a^	side reaction
2 [66]	NBS, AgNO_3_, MeCN/H_2_O, 0 °C, 5 min^b^	side reaction
3 [67]	PhI(OAc)_2_, MeCN/CH_2_Cl_2_/H_2_O, 50 °C, 18 h	no reaction^c^
4 [68]	Hg(ClO_4_)_2_, MeOH/CH_2_Cl_2_, rt, 2 h	side reaction
5 [69]	HgCl_2_,HgO, MeOH/H_2_O, 55 °C, 18 h^d^	no reaction
6 [70]	MeI, MeCN/H_2_O, reflux, 18 h	no reaction
7 [71]	*m*-CPBA^e^, MeCN, rt, 18 h, then 1 M HCl, reflux, 4 h	decomposition
8 [72]	SbCl_5_, CH_2_Cl_2_, 0 °C, 1 h	decomposition
9 [73]	I_2_, NaHCO_3_, acetone/H_2_O, 50 °C, 18 h	no reaction
10 [74]	CAN^f^, acetone/H_2_O, 50 °C, 18 h	no reaction
11 [75]	Chloramine T, MeOH/H_2_O, 70 °C, 18 h	no reaction

^a^Different solvent systems, such as acetone/H_2_O and DMSO were used, stoichiometry was varied and the reaction was run also at rt and for longer times (4 and 18 h) but in none of the cases was the desired product obtained. ^b^The reaction was also run at rt for 18 h, but the desired product was not obtained. ^c^Decomposition products were also observed. ^d^The MeCN/H_2_O solvent system was also used and the reaction was also run at rt and reflux, but in none of the cases was the desired product obtained. ^e^*m*-CPBA= *meta*-chloroperbenzoic acid. ^f^CAN = ceric ammonium nitrate.

**Scheme 4:**
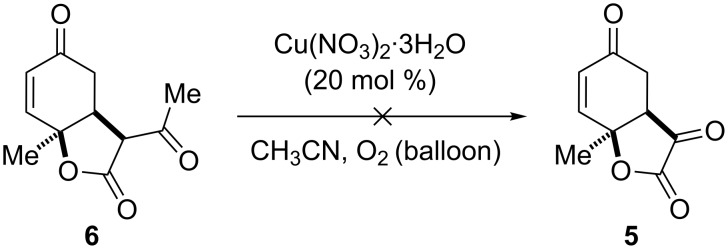
Attempted oxidative deacylation.

We attempted to synthesize **5** via an alternative route using Cu(II)-catalyzed aerobic oxidative deacylation [76] of the β-keto ester **6** (Scheme 4) [77]. The fact that this reaction also leads to decomposition of the starting material is cause for general concern about the feasibility of easily reaching the target substructure. In order to minimize the observed side reactions, we sought to apply the deprotection conditions to allylic alcohol **3**. However, both the use of NBS and HgCl_2_/HgO were unsuccessful.

**Scheme 5:**
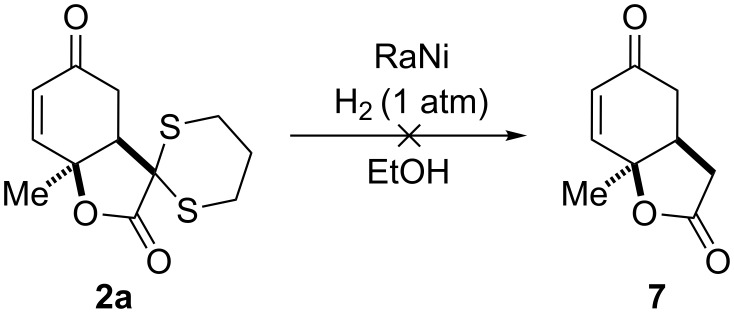
Attempted desulfurization with Raney nickel.

We further investigated the removal of the dithiane moiety via Raney nickel-promoted desulfurization (Scheme 5). To observe any substrate conversion, it was necessary to use a hydrogen atmosphere. Under those conditions, though the dithiane function was removed, degradation occurred.

## Conclusion

In conclusion, we have developed a desymmetrizing intramolecular conjugate addition of a tethered dithiane moiety to *para*-cresol-derived cyclohexadienones. The substrates are easily accessible from cheap starting materials and the reaction provides functionalized bicyclic lactones as a single diastereomer. The products of the reaction were able to undergo diastereoselective convex-facial additions. The carbonyl deprotection was unsuccessful and we hope that our efforts can serve as a cautionary tale for future synthetic planning involving related structures.

## Supporting Information

File 1 Experimental procedures, characterization data and copies of ^1^H and ^13^C NMR spectra for final compounds.

File 2 Crystallographic data.
