# Supplementary material for: Phosphazene-catalyzed desymmetrization of cyclohexadienones by dithiane addition
Source: Beilstein J Org Chem. 2017 Apr 24;13:762–7. doi: 10.3762/bjoc.13.75 (PMC5433211; doi:10.3762/bjoc.13.75)
Supplement: File 1 — Experimental procedures, characterization data and copies of 1H and 13C NMR spectra for final compounds. [file Beilstein_J_Org_Chem-13-762-s001.pdf]

Supporting Information

for

Phosphazene-catalyzed desymmetrization of cyclohexadienones by dithiane addition

Matthew A. Horwitz†, Elisabetta Massolo† and Jeffrey S. Johnson*

Address: Department of Chemistry, University of North Carolina at Chapel Hill, Chapel Hill, NC 27599-3290, USA

†These authors contributed equally to this work.

Email: Jeffrey S. Johnson - jsj@unc.edu

*Corresponding author

Experimental procedures, characterization data and copies of ^{1}H and ^{13}C NMR spectra for final compounds

Table of Contents	Page
General information (materials and methods)	S2
General procedure for substrate synthesis	S3
Characterization of substrates	S4–S5
General procedure for intramolecular conjugate addition of dithiane	S6
Characterization of products	S6–S8
Procedure for the Luche reduction of enone	S9
Characterization of Luche reduction product	S9
Procedure for 1,2-addition of $AlMe_3$	S9
Characterization of product of 1,2-addition of $AlMe_3$	S9
References	S10
Crude ^{1}H NMR spectra	S11–S18
^{1}H and ^{13}C NMR spectra of new compounds	S19–S50

General information:

Methods: Infrared (IR) spectra were obtained using an ASI ReactIR 1000 Fourier transform infrared spectrometer. Proton and carbon magnetic resonance spectra (^{1}H NMR, ^{13}C NMR, ^{19}F NMR and ^{31}P NMR) were recorded on a Bruker model DRX 400 or 600 (^{1}H NMR at 400 MHz or 600 MHz, ^{13}C NMR at 101 MHz or 151 MHz, or a Bruker AVANCE III-OneBay500 (^{13}C NMR at 235 MHz) spectrometer with solvent resonance as the internal standard (^{1}H NMR: $CDCl_3$ at 7.26 ppm and ^{13}C NMR: $CDCl_3$ at 77.0 ppm). ^{1}H NMR data are reported as follows: chemical shift, multiplicity (s = singlet, br-s = broad singlet, d = doublet, dd = doublet of doublet, t = triplet, td = triplet of doublet, m = multiplet), coupling constants (Hz), and integration. High resolution mass spectra were obtained with a Thermo Fisher Scientific Exactive or Finnigan^{TM} LTQ-ICR FT^{TM} (all samples prepared in methanol). Melting points were obtained using a Thomas Hoover UniMelt Capillary Melting Point Apparatus. Analytical thin layer chromatography was carried out using Whatman 0.25 mm silica gel 60 plates, Sorbent Technologies 0.20 mm Silica Gel TLC plates. Visualization was allowed by UV light, phosphomolybdic acid in ethanol, or aqueous ceric ammonium nitrate solution. Purification of the reaction products was carried out by using Siliaflash-P60 silica gel (40–63 μm) purchased from Silicycle. Yields refer to isolated yields after flash column chromatography. Since all results are the averages of two trials, the yields listed in the paper may not exactly match those listed below.

Materials: THF and DCM were purified by passing the solvent through a column of aluminum oxide under nitrogen. Dearomatization of phenol derivatives was carried out according to literature procedures.[1] 1,3-Dithiane-2-carboxylic acid was prepared according to literature procedure.[2] Phosphazene base P_2-*t*-Bu solution (~2.0 M in THF) and DMAP were purchased from Sigma-Aldrich and used as received. (Diacetoxyiodo)benzene, [bis(trifluoroacetoxy)iodo]benzene, and DCC were purchased from Oakwood Chemical and used as received.

General procedure for substrate synthesis:

The substrates were prepared through a two-step synthesis on gram scale.

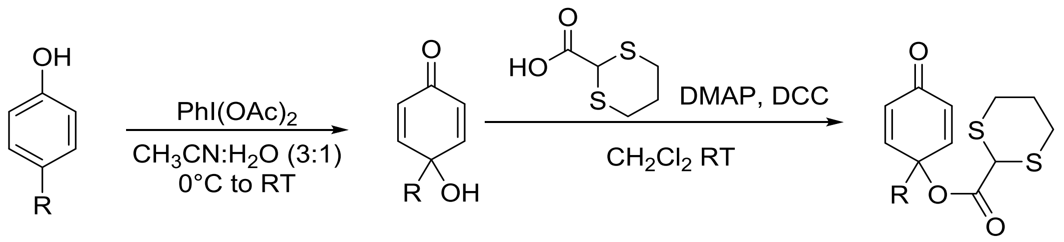

STEP 1 – Dearomatization of phenols[1]

The phenol (1 equiv) was dissolved in MeCN (3 mL for 1 mmol) and H_2O (1 mL for 1 mmol); the solution was cooled to 0 °C and $PhI(OAc)_2$ (1.1 equiv) was slowly added as a solid. The reaction mixture was allowed stirred at ambient temperature for 18 h. The mixture was diluted with EtOAc and washed with water and brine. The combined organic phases were dried over Na_2SO_4 and the solvent was removed under reduced pressure. The crude materials thusly obtained were purified using flash column chromatography on silica gel.

STEP 2 – DCC coupling between *para*-quinols and 1,3-dithiane-2-carboxylic acid

The desired *p*-quinols (1 equiv) and 1,3-dithiane-2-carboxylic acid (1.5 equiv) were dissolved in CH_2Cl_2 ($[quinol]_0$ = 1.0 M); 4-dimethylaminopyridine (DMAP) (1 equiv) was then added to the mixture. The reaction mixture was cooled to 0 °C and *N*,*N'*-dicyclohexylcarbodiimide (DCC) (1.1 equiv) was added. The reaction mixture was allowed to warm to rt and stirred for 18 h. After that period, the mixture was filtered through a short plug of silica gel and washed with CH_2Cl_2.The solvent was removed under reduced pressure. The crude materials thusly obtained were purified using flash column chromatography on silica gel.

Characterization of substrates:

1-Methyl-4-oxocyclohexa-2,5-dien-1-yl 1,3-dithiane-2-carboxylate (1a):

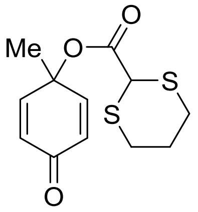

The title compound was obtained in 61% yield.

White solid, mp 88-90 °C; **^{1}H NMR** (600 MHz, $CDCl_3$) δ 6.96 (d, *J* = 10.2 Hz, 2H), 6.31 (d, *J* = 10.2 Hz, 2H), 4.12 (s, 1H), 3.43-3.39 (m, 2H), 2.61-2.57 (m, 2H), 2.19-2.14 (m, 1H), 2.06-1.99 (m, 1H), 1.62 (s, 3H); **^{13}C NMR** (151 MHz, $CDCl_3$) δ 184.8, 168.3, 148.6, 128.5, 75.3, 39.0, 26.4, 25.5, 24.8; **IR** (thin film) ν 2931, 1735, 1667, 1631, 1608, 1393, 1285, 1138, 1052, 857 cm^{-1}; **HRMS** (ESI): Calcd. For $C_{12}H_{14}NaO_3S_2^+$ ([M+Na$^+$]): 293.0277, found 293.0275; **TLC** (1:4 EtOAc/hexanes): R_f = 0.33.

1-Ethyl-4-oxocyclohexa-2,5-dien-1-yl 1,3-dithiane-2-carboxylate (1b):

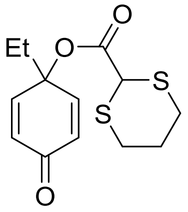

The title compound was obtained in 49% yield.

Yellow oil; **^{1}H NMR** (600 MHz, $CDCl_3$) δ 6.88 (d, *J* = 10.2 Hz, 2H), 6.36 (d, *J* = 10.2 Hz, 2H), 4.14 (s, 1H), 3.40 (ddd, *J* = 14.3, 12.2, 2.5 Hz, 1H), 2.60-2.57 (m, 1H), 2.18-2.14 (m 1H), 2.06-1.99 (m, 1H), 1.94 (q, *J* = 7.5 Hz, 1H), 0.94 (t, *J* = 7.5 Hz, 2H). **^{13}C NMR** (151 MHz, $CDCl_3$) δ 185.1, 168.3, 147.6, 129.6, 78.4, 39.2, 32.2, 25.5, 24.8, 7.7; **IR** (thin film) ν 2935, 1736, 1667, 1631, 1282, 1138, 1063, 995, 915, 853 cm^{-1}; **HRMS** (ESI): Calcd. For $C_{13}H_{16}NaO_3S_2^+$ ([M+Na$^+$]): 307.0433, found 307.0428; **TLC** (1:4 EtOAc/hexanes): R_f = 0.38.

1-(3-Methoxy-3-oxopropyl)-4-oxocyclohexa-2,5-dien-1-yl 1,3-dithiane-2-carboxylate (1c):

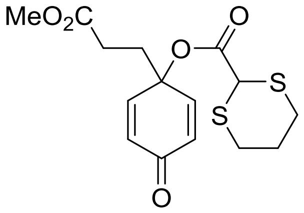

The title compound was obtained in 49% yield.

Light yellow solid, mp 79-80 °C; **^{1}H NMR** (600 MHz, $CDCl_3$) δ 6.85 (d, *J* = 10.2 Hz, 2H), 6.34 (d, *J* = 10.1 Hz, 2H), 4.10 (s, 1H), 3.66 (s, 3H), 3.39-3.34 (m, 2H), 2.58-2.54 (m, 2H), 2.36-2.33 (m, 2H), 2.26-2.23 (m, 2H), 2.16-2.12 (m, 1H), 2.04-1.97 (m, 1H). **^{13}C NMR** (151 MHz, $CDCl_3$) δ 184.6, 172.5, 168.0, 146.7, 129.9, 52.0, 39.0, 33.8, 28.1, 25.5, 24.8; **IR** (thin film) ν 2949, 2360, 1734, 1670, 1654, 1521, 1473, 1281, 1136, 990 cm^{-1}; **HRMS** (ESI): Calcd. For $C_{15}H_{18}NaO_5S_2^+$ ([M+Na$^+$]): 365.0488, found 365.0478; **TLC** (1:4 EtOAc/hexanes): R_f = 0.25.

1-(2-((*tert*-Butyldimethylsilyl)oxy)ethyl)-4-oxocyclohexa-2,5-dien-1-yl 1,3-dithiane-2-carboxylate (1d):

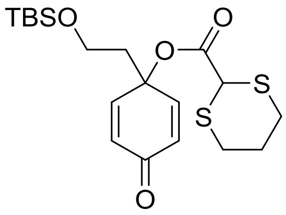

The title compound was obtained in 55% yield.

Clear oil; **^{1}H NMR** (600 MHz, $CDCl_3$) δ 6.98 (d, *J* = 10.2 Hz, 2H), 6.29 (d, *J* = 10.2 Hz, 2H), 4.11 (s, 1H), 3.75 (t, *J* = 9.0 Hz, 2H), 3.42-3.35 (m, 2H), 2.59-2.55 (m, 2H), 2.18-2.16 (m, 1H), 2.08 (t, *J* = 6.1 Hz, 2H), 2.04-1.95 (m, 1H), 0.87 (s, 9H), 0.03 (s, 6H). **^{13}C NMR** (151 MHz, $CDCl_3$) δ 185.1, 168.1, 147.9, 128.6, 57.7, 42.7,

39.2, 25.8, 25.5, 24.8, 18.1; **IR** (thin film) ν 2929, 2855, 1739, 1670, 1635, 1508, 1472, 1256, 1097, 838 cm^{-1}; **HRMS** (ESI): Calcd. For $C_{19}H_{30}NaO_4S_2Si^+$ ([M+Na^+]): 437.1247, found 437.1233; **TLC** (1:4 EtOAc/hexanes): R_f = 0.32.

4-Oxo-[1,1'-biphenyl]-1(4H)-yl 1,3-dithiane-2-carboxylate (1e):

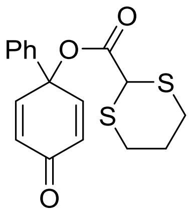

The title compound was obtained in 18% yield.

Orange solid, mp 113-114 °C; **^{1}H NMR** (600 MHz, $CDCl_3$) δ 7.41-7.46 (m, 2H), 7.41-7.34 (m, 3H), 7.06 (d, *J* = 10.1 Hz, 2H), 6.38 (d, *J* = 10.1 Hz, 2H), 4.26 (s, 1H), 3.37 (td, *J* = 12.0, 6.1 Hz, 2H), 2.60-2.57 (m, 2H), 2.15-2.12 (m, 1H), 2.06-1.99 (m, 1H).**^{13}C NMR** (151 MHz, $CDCl_3$) δ 185.3, 167.7, 146.8, 136.2, 129.2, 129.0, 128.5, 125.2, 39.6, 25.6, 24.8; **IR** (thin film) ν 2920, 2360, 1739, 1669, 1277, 1126, 994, 849, 698 cm^{-1}; **HRMS** (ESI): Calcd. For $C_{17}H_{16}NaO_3S_2^+$ ([M+Na^+]): 355.0433, found 355.0425; **TLC** (1:4 EtOAc/hexanes): R_f = 0.44.

1-(2-((*tert*-Butoxycarbonyl)amino)ethyl)-4-oxocyclohexa-2,5-dien-1-yl 1,3-dithiane-2-carboxylate (1f):

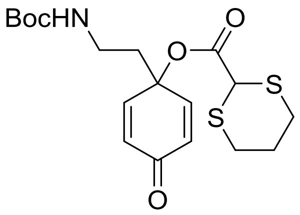

The title compound was obtained in 17% yield.

White solid, mp 107-109 °C; **^{1}H NMR** (600 MHz, $CDCl_3$) δ 6.91 (d, *J* = 10.1 Hz, 2H), 6.32 (d, *J* = 10.1 Hz, 2H), 4.68 (bs, 1H), 4.10 (s, 1H), 3.22-3.21 (m, 2H), 3.22-3.21 (m, 2H), 2.58-2.54 (m, 2H), 2.14-2.11 (m, 1H), 2.08-2.05 (m, 2H), 2.03-1.95 (m, 1H), 1.41 (s, 9H). **^{13}C NMR** (151 MHz, $CDCl_3$) δ 184.6, 168.0, 155.6, 147.0, 129.5, 79.7, 39.6, 39.3, 35.7, 33.9, 28.4, 25.6, 24.8; **IR** (thin film) ν 3354, 2975, 2929, 1738, 1668, 1517, 1366, 1274, 1169, 859 cm^{-1}; **HRMS** (ESI): Calcd. For $C_{18}H_{25}NNaO_5S_2^+$ ([M+Na^+]): 422.1066, found 422.1054; **TLC** (1:4 EtOAc/hexanes): R_f = 0.24.

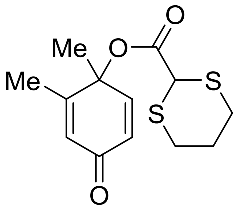

1,2-Dimethyl-4-oxocyclohexa-2,5-dien-1-yl 1,3-dithiane-2-carboxylate (1g):

The title compound was obtained in 67% yield.

White solid, mp 120-121 °C; **^{1}H NMR** (600 MHz, $CDCl_3$) δ 6.91 (d, *J* = 10.2 Hz, 1H), 6.29 (dd, *J* = 9.9, 2.4 Hz, 1H), 6.16 (s, 1H), 4.13 (s, 1H), 3.48-3.36 (m, 2H), 2.61-2.56 (m, 2H), 2.20-2.15 (m, 1H), 2.07-1.99 (m, 1H), 2.04 (d, *J* = 1.2 Hz, 3H), 1.57 (s, 3H); **^{13}C NMR** (151 MHz, $CDCl_3$) δ 185.3, 168.0, 158.8, 149.4, 128.1, 126.8, 38.4, 26.4, 25.3, 25.3, 24.8, 17.8; ; **IR** (thin film) ν 2933, 1734, 1668, 1613, 1433, 1391, 1293, 1134, 1056, 885 cm^{-1}; **HRMS** (ESI): Calcd. For $C_{13}H_{16}NaO_3S_2^+$ ([M+Na^+]): 307.0433, found 307.0431; **TLC** (1:4 EtOAc/hexanes): R_f = 0.25.

General procedure for intramolecular conjugate addition of dithiane:

A flame-dried 1 dram vial was charged sequentially with the dithiane-tethered cyclohexadienone (0.1 mmol, 1.0 equiv), followed by THF (1.0 mL), and then P2-*t*-Bu phosphazene (0.02 mmol, 20 mol %). The reaction was stirred at room temperature for 30 min. The reaction was quenched with saturated ammonium chloride, and the layers were separated. The aqueous layer was extracted three times with ethyl acetate, and then the combined organic phases were dried with sodium sulfate, and concentrated in vacuo. The crude materials thusly obtained were purified using flash column chromatography on silica gel using a hexane/EtOAc system (typically EtOAc/hexanes 1:9).

Characterization of products:

7a-Methyl-3a,7a-dihydro-2*H*-spiro[benzofuran-3,2'-[1,3]dithiane]-2,5(4*H*)-dione (2a):

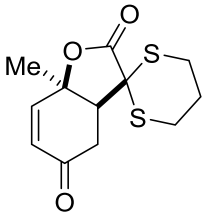

The title compound was prepared by the general procedure. White solid, mp 185-187 °C; **^{1}H NMR** (600 MHz, $CDCl_3$) δ 6.79 (dd, *J* = 10.5 Hz, 1.8 Hz, 1H), 6.10 (d, *J* = 10.2 Hz, 1H), 3.99-3.94 (m, 1H), 3.42-3.37 (m, 1H), 2.99 (d, *J* = 18.6 Hz, 1H), 2.87 (d, *J* = 7.2 Hz, 1H), 2.69 (dd, *J* = 18.6 Hz, 7.8 Hz, 1H), 2.63-2.61 (m, 2H), 2.17-2.13 (m, 1H), 1.87-1.80 (m, 1H), 1.68 (s, 3H); **^{13}C NMR** (151 MHz, $CDCl_3$) δ 193.4, 171.9, 147.7, 130.0, 78.8, 52.5, 49.5, 33.4, 27.4, 26.6, 25.9, 23.6; ; **IR** (thin film) ν 2920, 1761, 1681, 1275, 1194, 1104, 1067, 972, 787, 734 cm^{-1}; **HRMS** (ESI): Calcd. For $C_{12}H_{15}O_3S_2^+$ ([$M+H^+$]): 271.0457, found 271.0457: **TLC** (2:8 EtOAc/hexanes): R_f = 0.13.

7a-Ethyl-3a,7a-dihydro-2*H*-spiro[benzofuran-3,2'-[1,3]dithiane]-2,5(4*H*)-dione (2b):

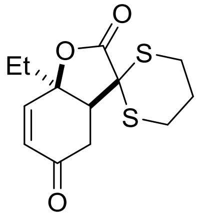

The title compound was prepared by the general procedure. White solid, mp 109-110 °C; **^{1}H NMR** (400 MHz, $CDCl_3$) δ 6.80 (d, *J* = 10.5 Hz, 1H), 6.18 (d, *J* = 10.6 Hz, 1H), 4.02-3.94 (m, 1H), 3.45-3.38 (m, 1H), 3.01 (d, *J* = 18.9 Hz, 1H), 2.89 (d, *J* = 8.0 Hz, 1H), 2.68-2.61 (m, 3H), 2.20-2.13 (m, 1H), 2.04-1.98 (m, 1H), 1.94-1.89 (m, 1H), 1.87-1.81 (m, 1H), 1.09 (t, *J* = 5.0 Hz, 3H); **^{13}C NMR** (151 MHz, $CDCl_3$) δ 193.8, 172.0, 147.0, 130.8, 81.2, 50.1, 49.7, 33.8, 32.8, 27.3, 26.0, 23.7, 7.8 **IR** (thin film) ν 3425, 2970, 1757, 1681, 1388, 1223, 1187, 1116, 978, 9321 cm^{-1}; **HRMS** (ESI): Calcd. For $C_{13}H_{16}NaO_3S_2^+$ ([$M+Na^+$]): 307.0433, found 307.0433; **TLC** (2:8 EtOAc/hexanes): R_f = 0.28.

Methyl 3-(2,5-dioxo-4,5-dihydro-2*H*-spiro[benzofuran-3,2'-[1,3]dithian]-7a(3a*H*)-yl)propanoate (2c): The title compound was prepared by the general procedure. White solid, mp 114-115 °C; **^{1}H NMR** (600 MHz, $CDCl_3$) δ 6.78 (dd, J = 10.5,1.6 Hz, 1H), 6.17 (d, *J* = 10.6 Hz, 1H), 3.98-3.93 (m, 1H), 3.72 (s, 3H), 3.42-3.37 (m, 1H), 3.00 (d, *J* = 18.9 Hz, 1H), 2.89 (d, *J* = 7.7 Hz, 1H), 2.69 (dd, *J* = 18.8, 7.8 Hz, 1H), 2.64-2.61 (m, 2H), 2.59-2.51 (m, 2H), 2.35-2.30 (m, 1H), 2.25-2.20 (m, 1H), 2.18-2.14 (m, 1H), 1.88-1.81 (m, 1H); **^{13}C NMR** (151 MHz, $CDCl_3$) δ 193.3, 172.5, 171.6, 146.0, 131.1, 79.8, 52.2, 50.5, 49.3, 34.4, 33.4, 28.0, 27.3, 26.0, 23.6; **IR** (thin film) ν 2923, 2852, 1760, 1735, 1685, 1435, 1166, 1079, 972, 932 cm^{-1}; **HRMS** (ESI): Calcd. For $C_{15}H_{19}O_5S_2Si^+$ ([M+H$^+$]): 343.0668, found 343.0663; **TLC** (3:7 EtOAc/hexanes): R_f = 0.14.

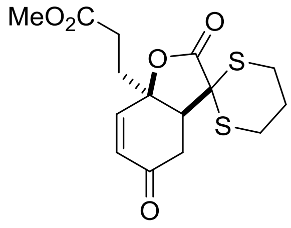

7a-(2-((*tert*-Butyldimethylsilyl)oxy)ethyl)-3a,7a-dihydro-2*H*-spiro[benzofuran-3,2'-[1,3]dithiane]-2,5(4*H*)-dione (2d):

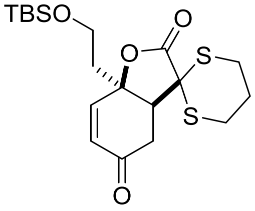

The title compound was prepared by the general procedure. White solid, mp 135-137 °C; **^{1}H NMR** (600 MHz, $CDCl_3$) δ 6.78 (d, *J* = 10.5 Hz, 1H), 6.15 (d, *J* = 10.5 Hz, 1H), 3.97 (td, *J* = 13.6, 2.4 Hz, 1H), 3.89-3.86 (m, 1H), 3.80-3.76 (m, 1H), 3.43-3.93 (m, 1H), 3.35 (d, *J* = 7.8 Hz, 1H), 2.96 (d, *J* = 18.8 Hz, 1H), 2.81 (dd, *J* = 18.8, 7.7 Hz, 1H), 2.63-2.60 (m, 1H), 2.18-2.14 (m, 1H),2.08-2.04 (m, 1H), 2.88-2.81 (m, 1H), 0.90 (s, 9H), 0.09 (s, 3H), 0.08 (s, 3H); **^{13}C NMR** (151 MHz, $CDCl_3$) δ 194.3, 172.2, 147.4, 130.5, 80.7, 57.7, 50.0, 49.5, 41.6, 33.5, 27.3, 26.0, 25.8, 23.7, 18.1, -5.5, -5.5; **IR** (thin film) ν 23434, 2953, 1761, 1642, 1407, 1248, 1182, 1095, 778 cm^{-1}; **HRMS** (ESI): Calcd. For $C_{19}H_{31}O_4S_2Si^+$ ([M+H$^+$]): 415.1428, found 415.1420; **TLC** (2:8 EtOAc/hexanes): R_f = 0.37.

7a-Phenyl-3a,7a-dihydro-2*H*-spiro[benzofuran-3,2'-[1,3]dithiane]-2,5(4*H*)-dione (2e):

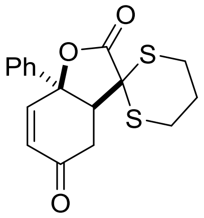

The title compound was prepared by the general procedure. White solid, mp 171-172 °C; **^{1}H NMR** (600 MHz, $CDCl_3$) δ 7.46-7.42 (m, 5H), 6.87 (d, *J* = 10.2 Hz, 1H), 6.40 (d, *J* = 10.2 Hz, 1H), 4.02 (t, *J* = 13.2 Hz, 1H), 3.50 (t, *J* = 14.4 Hz, 1H), 3.03 (d, *J* = 7.2 Hz, 1H), 2.99 (d, *J* = 18.6 Hz, 1H), 2.72-2.64 (m, 3H), 2.20 (m, 1H), 1.87 (q, *J* = 13.2 Hz, 1H); **^{13}C NMR** (151 MHz, $CDCl_3$) δ 193.8, 172.0, 145.3, 138.8, 131.5, 129.3, 129.2, 124.6, 81.7, 54.6, 49.4, 33.0, 27.4, 26.0, 23.7; **IR** (thin film) ν 22971, 2361, 1769, 1684, 1540, 1507, 1224, 1170, 997, 799 cm^{-1}; **HRMS** (ESI): Calcd. For $C_{17}H_{17}O_3S_2^+$ ([M+H$^+$]): 333.0614, found 333.0608; **TLC** (3:7 EtOAc/hexanes): R_f = 0.38.

tert-Butyl 2,5-dioxohexahydro-2H-spiro[furo[2,3-d]indole-3,2'-[1,3]dithiane]-7(3aH)-carboxylate (2f):

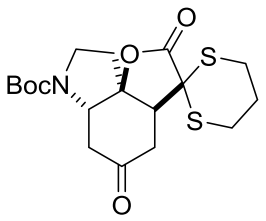

The title compound was prepared by the general procedure. Two rotamers were observed in a 56:44 ratio in the ^{1}H NMR spectrum. White solid, mp 196-197 °C; **^{1}H NMR** (600 MHz, $CDCl_3$) δ 4.32-4.04 (m, 1H for the minor rotamer), 4.28-4.25 (m, 1H for the major rotamer) 3.77-3.58 (m, 4H), 3.19 (dd, *J* = 17.2, 5.8 Hz, 1H for the minor rotamer), 3.02 (dd, *J* = 17.1, 5.7 Hz, 1H for the major rotamer)., 2.80-2.75 (m, 1H), 2.75-2.73 (m, 4H), 2.63-2.59 (m, 1H), 2.32-2.26 (m, 1H), 2.24-2.19 (m, 1H), 2.10-1.99 (m, 1H), 1.96-1.89 (m, 1H), 1.48 (s, 9H); **^{13}C NMR** (151 MHz, $CDCl_3$) δ 205.0, 204.7, 171.2, 153.8, 153.5, 88.7, 88.0, 80.7, 60.6, 60.5, 49.3, 48.3, 48.2, 44.4, 44.0, 43.6, 42.9, 37.3, 37.2, 35.7, 34.8, 28.5, 28.4, 27.6, 27.5, 26.4, 26.3, 23.8; **IR** (thin film) ν 2974, 2927, 1764, 1721, 1691, 1399, 1249, 1174, 1136 975 cm^{-1}; **HRMS** (ESI): Calcd. For $C_{18}H_{25}NNaO_5S_2$ ($[M+Na^+]$): 422.1067, found 422.1077; **TLC** (1:1 EtOAc/hexanes): R_f = 0.50.

7,7a-Dimethyl-3a,7a-dihydro-2H-spiro[benzofuran-3,2'-[1,3]dithiane]-2,5(4H)-dione (2g):

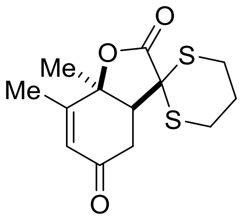

The title compound was prepared by the general procedure. Impurity present in the δ 2.73-2.67 muliplet makes integral appear as 3H. Orange-brown solid, mp 169-179 °C; **^{1}H NMR** (600 MHz, $CDCl_3$) δ 5.96 (s, 1H), 3.96 (t, *J* = 13.8 Hz, 1H), 3.35 (t, *J* = 13.8 Hz, 1H), 2.98 (d, *J* = 19.2 Hz, 1H), 2.868 (d, *J* = 9.0 Hz, 1H), 2.73-2.67 (m, 1H), 2.63-2.60 (m, 2H), 2.17-2.14 (m, 1H), 2.09 (s, 3H), 1.83 (q, *J* = 13.2 Hz, 1H), 1.69 (s, 3H); **^{13}C NMR** (151 MHz, $CDCl_3$) δ 193.2, 171.9, 159.3, 128.5, 81.0, 53.2, 49.5, 33.1, 27.1, 26.1, 25.7, 23.7, 18.4; **IR** (thin film) ν 2920, 1266, 1760, 1671, 1425, 1228, 1190, 1098, 970, 935 cm^{-1}; **HRMS** (ESI): Calcd. For $C_{13}H_{17}O_3S_2$ ($[M+Na^+]$): 285.0614, found 285.0611; **TLC** (2:8 EtOAc/hexanes): R_f = 0.13.

Procedure for the preparation of 5-hydroxy-7a-methyl-3a,4,5,7a-tetrahydro-2*H*-spiro[benzofuran-3,2'-[1,3]dithian]-2-one (3) via Luche reduction of enone 2a: $CeCl_3 \cdot 7H_2O$ (1.2 equiv) and $NaBH_4$ (5.3 equiv) were added to a solution of the substrate (**2a**) in MeOH (0.2 M) at −10 °C. The reaction mixture was stirred at the same temperature for 40 min. After this period, it was quenched with 1 M HCl and extracted with EtOAc three times. The combined organic phases were dried over Na_2SO_4, filtered, and concentrated under reduced pressure. The crude residue was purified by flash column chromatography on silica gel using EtOAc/hexanes 3:7 as the eluent.

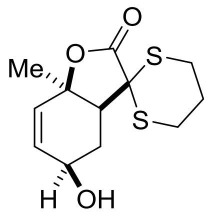

White solid, mp 97-99 °C; **^{1}H NMR** (600 MHz, $CDCl_3$) δ 5.95 (d, *J* = 10.2 Hz, 1H), 5.79 (d, *J* = 10.2 Hz, 1H), 4.20-4.16 (br, 1H), 4.00 (t, *J* = 13.8 Hz, 1H), 3.62 (t, *J* = 18.3 Hz, 1H), 2.68-2.64 (m, 2H), 2.46-2.43 (d, *J* = 12.0, 5.4 Hz, 1H), 2.41-2.37 (m, 1H), 2.22-2.18 (m, 1H), 1.98-1.90 (m, 1H), 1.86 (d, *J* = 7.2 Hz, 1H), 1.77-1.70 (m, 1H), 1.67 (s, 3H); **^{13}C NMR** (151 MHz, $CDCl_3$) δ 172.6, 134.7, 129.6, 65.4, 49.9, 49.8, 49.8, 31.3, 29.0, 28.2, 26.5, 24.3; **IR** (thin film) ν 3035, 2926, 1752, 1423, 1276 1189, 1148, 1061, 956, 732 cm^{-1}; **HRMS** (ESI): Calcd. For $C_{12}H_{17}O_3S_2$ ([M+Na$^+$]): 273.0614, found 273.0610; **TLC** (6:4 EtOAc/hexanes): R_f = 0.34.

Procedure for the preparation of 5-hydroxy-5,7a-dimethyl-3a,4,5,7a-tetrahydro-2*H*-spiro[benzofuran-3,2'-[1,3]dithian]-2-one (4) via 1,2-addition of $AlMe_3$: A solution of the substrate (**2a**) in dry CH_2Cl_2 was added to a solution of $AlMe_3$ (2 M in toluene, 4 equiv) in dry CH_2Cl_2 at 0 °C. The reaction mixture was allowed to reach room temperature and kept under stirring for 3 h. After this period, it was quenched with MeOH and extracted with CH_2Cl_2 and EtOAc. The combined organic phases were dried over anhydrous Na_2SO_4 and the solvent was removed under reduced pressure.

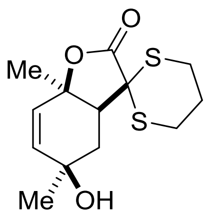

Light yellow solid, mp 179-180 °C; **^{1}H NMR** (600 MHz, $CDCl_3$) δ 5.91 (dd, *J* = 10.1, 1.4 Hz, 1H), 5.67 (d, *J* = 10.1 Hz, 1H), 4.06-4.01 (m, 1H), 3.62-3.57 (m, 1H), 2.71-2.65 (m, 3H), 2.50 (dd, *J* = 12.7, 5.6 Hz, 1H), 2.23-2.16 (m, 2H), 1.95-1.91 (m, 2H), 1.70 (s, 3H), 1.28 (s, 3H); **^{13}C NMR** (151 MHz, $CDCl_3$) δ 172.7, 137.9, 128.0, 79.1, 68.9, 50.0, 36.7, 29.3, 28.3, 26.5, 26.4, 24.4; **IR** (thin film) ν 3433, 2960, 2088, 1752, 1643, 1373, 1280, 1193, 1151, 1055 cm^{-1}; **HRMS** (ESI): Calcd. For $C_{13}H_{18}NaO_3S_2$ ([M+Na$^+$]): 309.0590, found 309.0593; **TLC** (4:6 EtOAc/hexanes): R_f = 0.43.

References:

1. a) Felpin, F.-X. *Tetrahedron Lett.* **2007**, *48*, 409. b) McKillop, A.; McLaren, L.; Taylor, R. J. K. *J. Chem. Soc., Perkin Trans. 1*, **1994**, *15*, 2047. c) Jepsen, T. H.; Jensen, A. A.; Lund, M. H.; Glibstrup, E.; Kristensen, J. L. *ACS Med. Chem. Lett.* **2014**, *5*, 766. d) Canesi, S.; Bouchu, D.; Ciufolini, M. A. *Org. Lett.* **2005**, *7*, 175. e) Brown, P. D.; Willis, A. C.; Sherburn, M. S.; Lawrence, A. L. *Org. Lett.* **2012**, *14*, 4537.
2. Kondoh, A.; Oishi, M.; Takeda, T.; Terada, M. *Angew. Chem., Int. Ed.* **2015**, *54*, 15836.

Crude ¹H NMR spectra for the desymmetrization reaction:

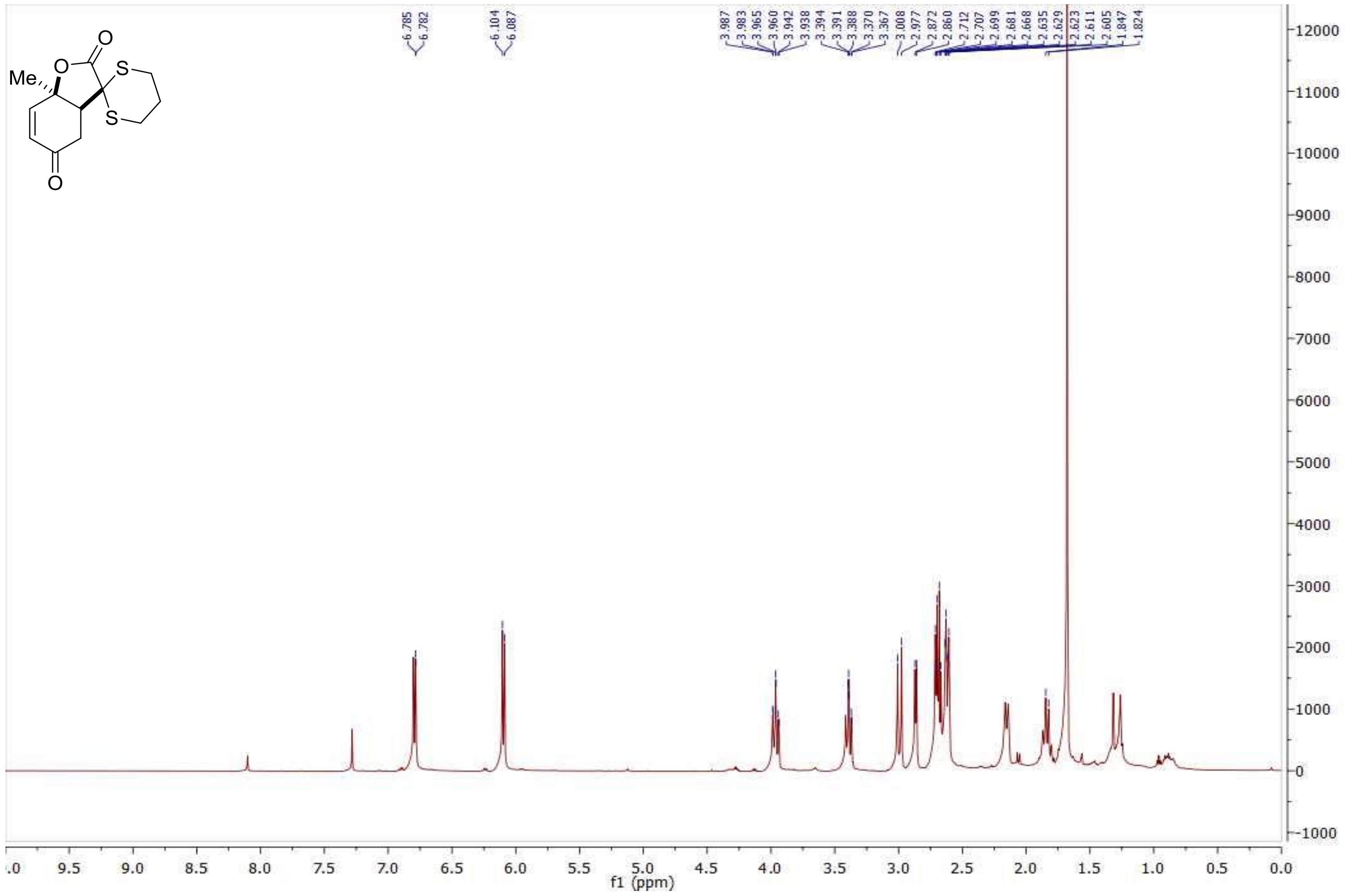

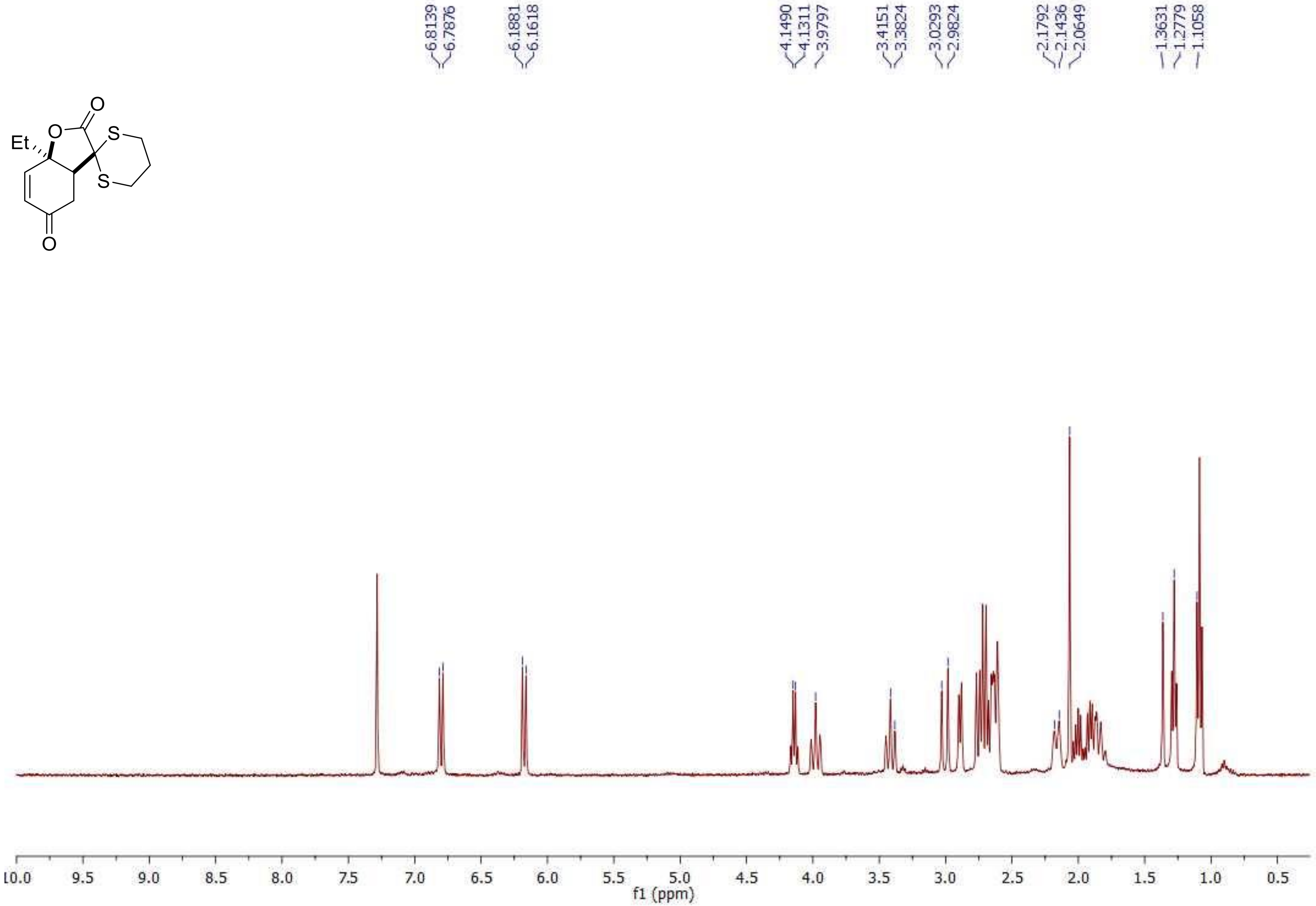

6.8139
6.7876
6.1881
6.1618
4.1490
4.1311
3.9797
3.4151
3.3824
3.0293
2.9824
2.1792
2.1436
2.0649
1.3631
1.2779
1.1058
10.0 9.5 9.0 8.5 8.0 7.5 7.0 6.5 6.0 5.5 5.0 4.5 4.0 3.5 3.0 2.5 2.0 1.5 1.0 0.5
f1 (ppm)

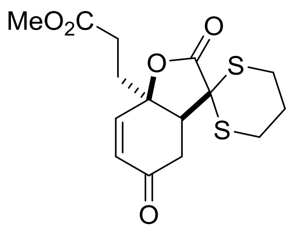
MeO2C
O
O
S
S
O

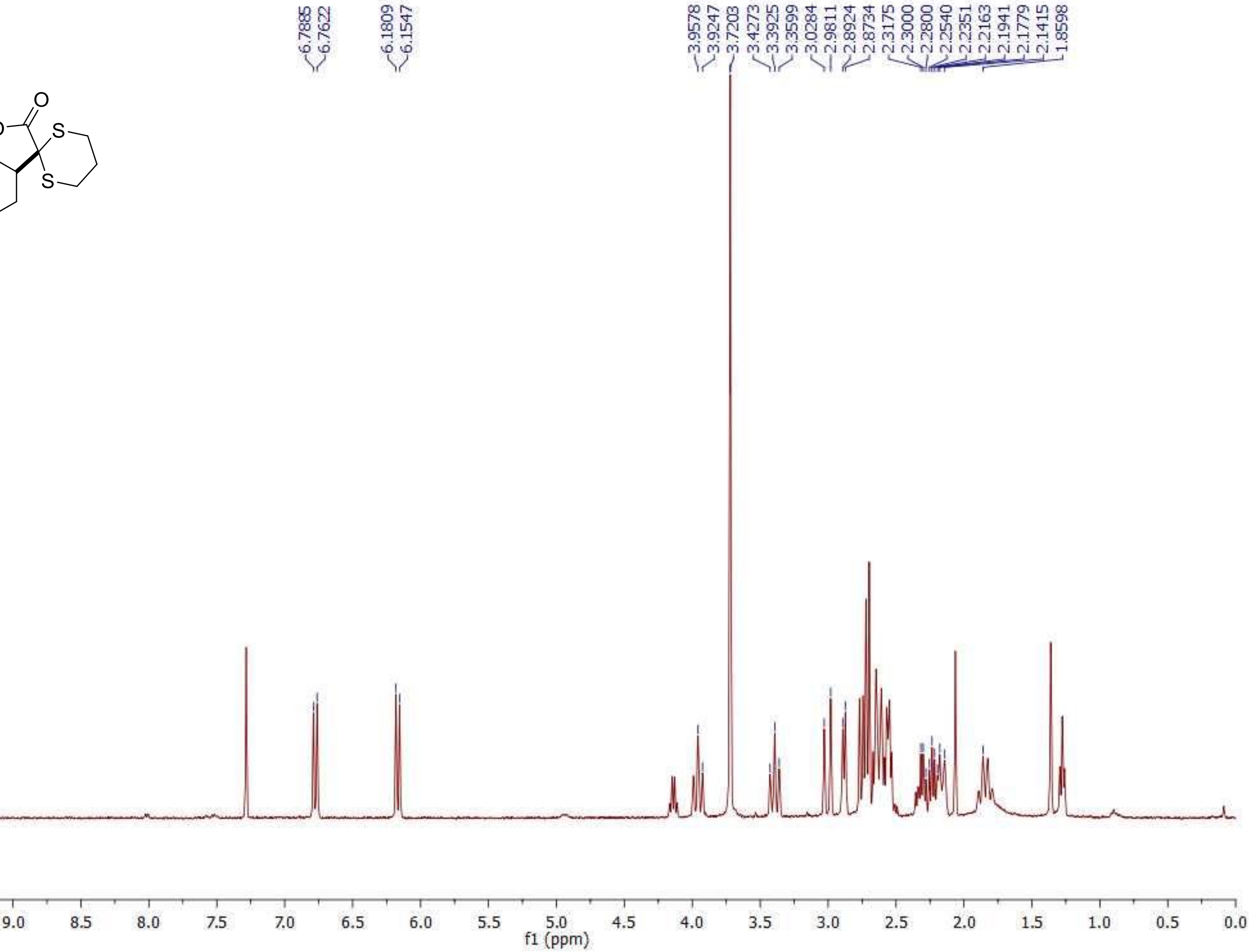
6.7885
6.7622
6.1809
6.1547
3.9578
3.9247
3.7203
3.4273
3.3925
3.3599
3.0284
2.9811
2.8924
2.8734
2.3175
2.3000
2.2800
2.2540
2.2351
2.2163
2.1941
2.1779
2.1415
1.8598
10.0 9.5 9.0 8.5 8.0 7.5 7.0 6.5 6.0 5.5 5.0 4.5 4.0 3.5 3.0 2.5 2.0 1.5 1.0 0.5 0.0
f1 (ppm)

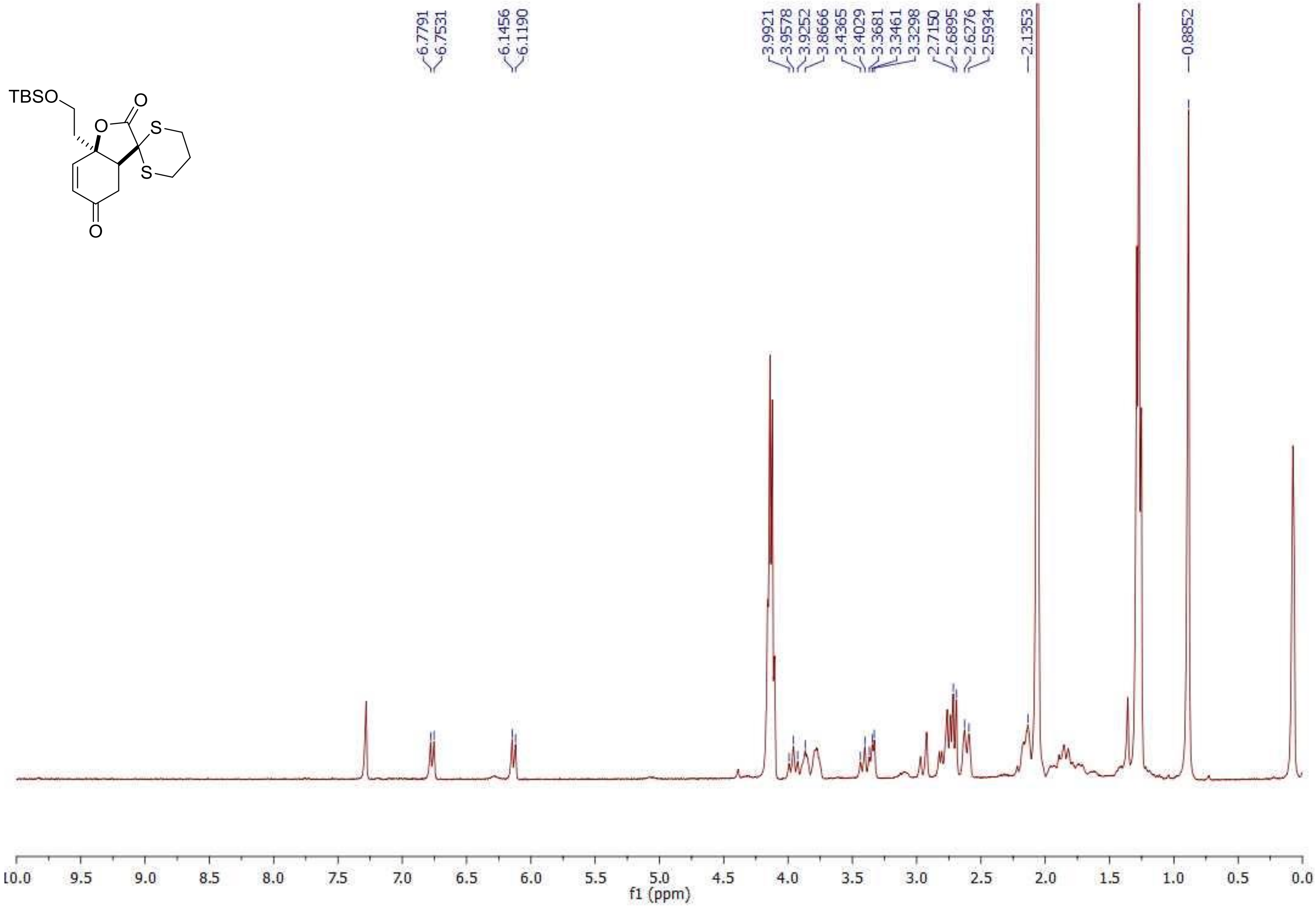
TBSO
O
O
S
S
O
6.7791
6.7531
6.1456
6.1190
3.9921
3.9578
3.9252
3.8666
3.4365
3.4029
3.3681
3.3461
3.3298
2.7150
2.6895
2.6276
2.5934
2.1353
0.8852
10.0 9.5 9.0 8.5 8.0 7.5 7.0 6.5 6.0 5.5 5.0 4.5 4.0 3.5 3.0 2.5 2.0 1.5 1.0 0.5 0.0
f1 (ppm)

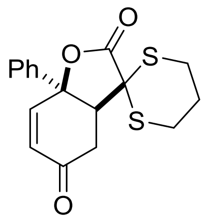
O
O
Ph
S
S
O

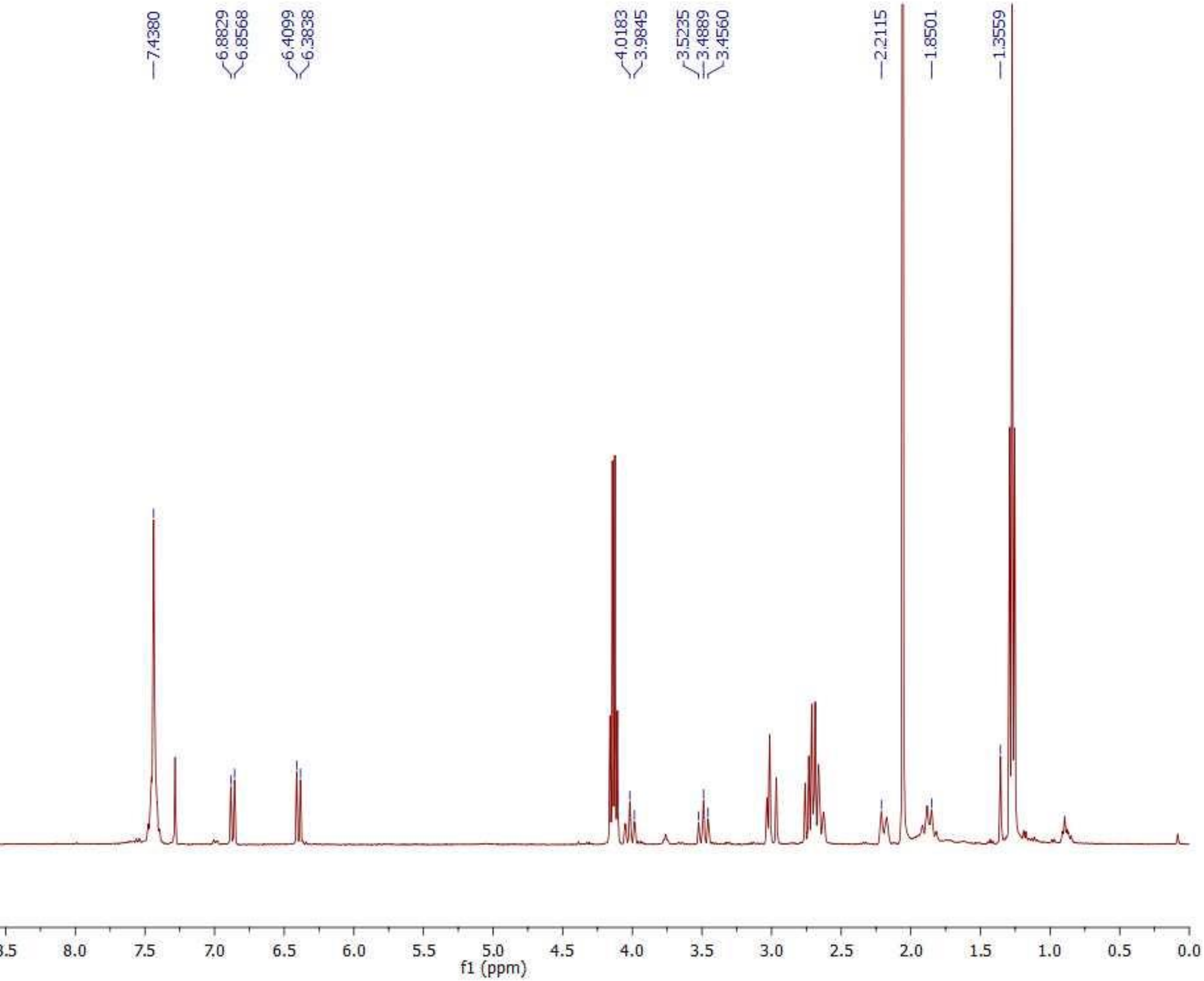
7.4380
6.8829
6.8568
6.4099
6.3838
4.0183
3.9845
3.5235
3.4889
3.4560
2.2115
1.8501
1.3559
10.0 9.5 9.0 8.5 8.0 7.5 7.0 6.5 6.0 5.5 5.0 4.5 4.0 3.5 3.0 2.5 2.0 1.5 1.0 0.5 0.0
f1 (ppm)

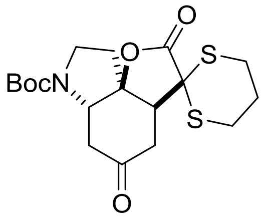

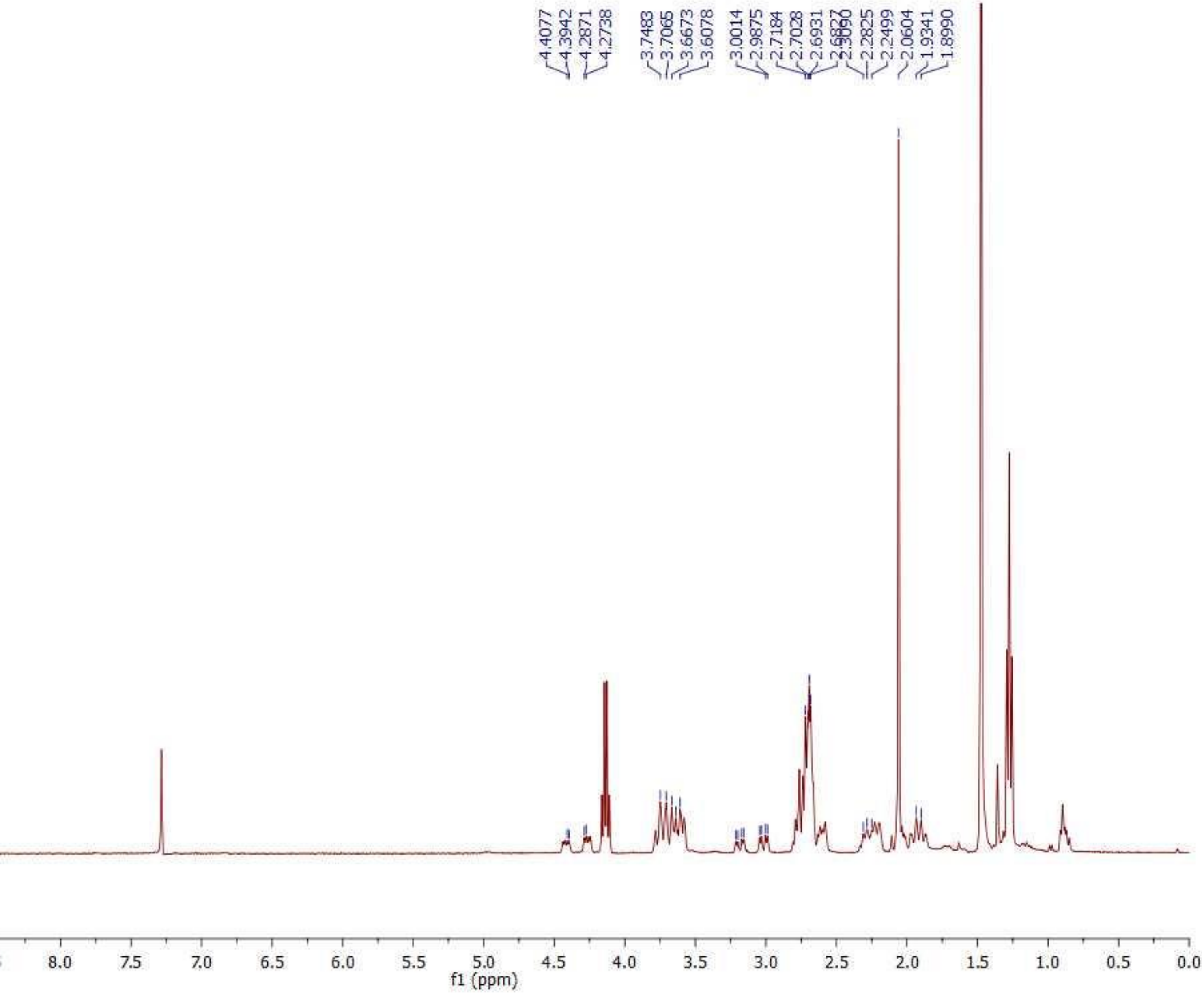
4.4077
4.3942
4.2871
4.2738
3.7483
3.7065
3.6673
3.6078
3.0014
2.9875
2.7184
2.7028
2.6931
2.6827
2.3090
2.2825
2.2499
2.0604
1.9341
1.8990
10.0
9.5
9.0
8.5
8.0
7.5
7.0
6.5
6.0
5.5
5.0
4.5
4.0
3.5
3.0
2.5
2.0
1.5
1.0
0.5
0.0
f1 (ppm)

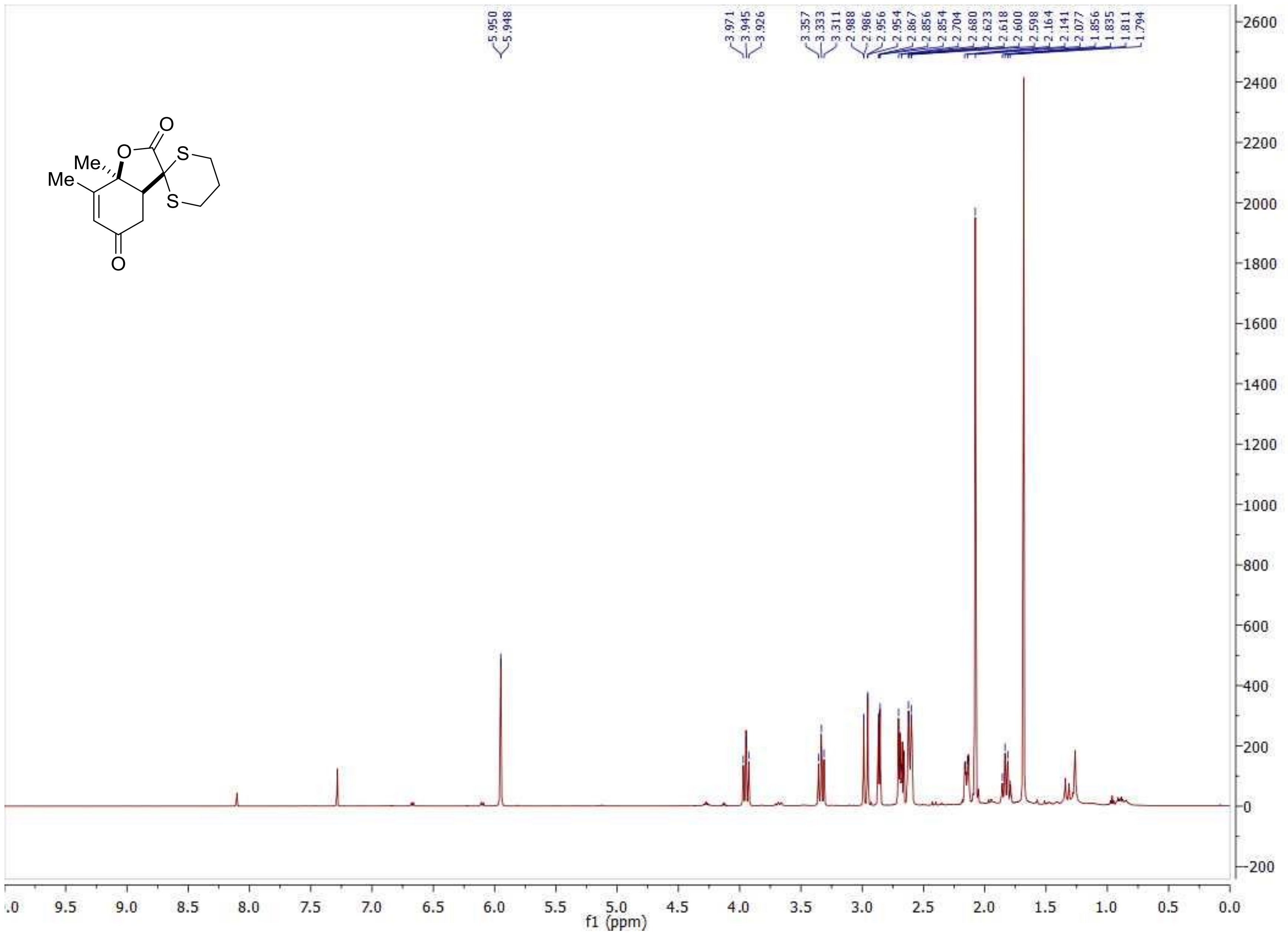

Me
Me
O
O
S
S
O
5.950
5.948
3.971
3.945
3.926
3.357
3.333
3.311
2.988
2.986
2.956
2.954
2.867
2.856
2.854
2.704
2.680
2.623
2.618
2.600
2.598
2.164
2.141
2.077
1.856
1.835
1.811
1.794
f1 (ppm)

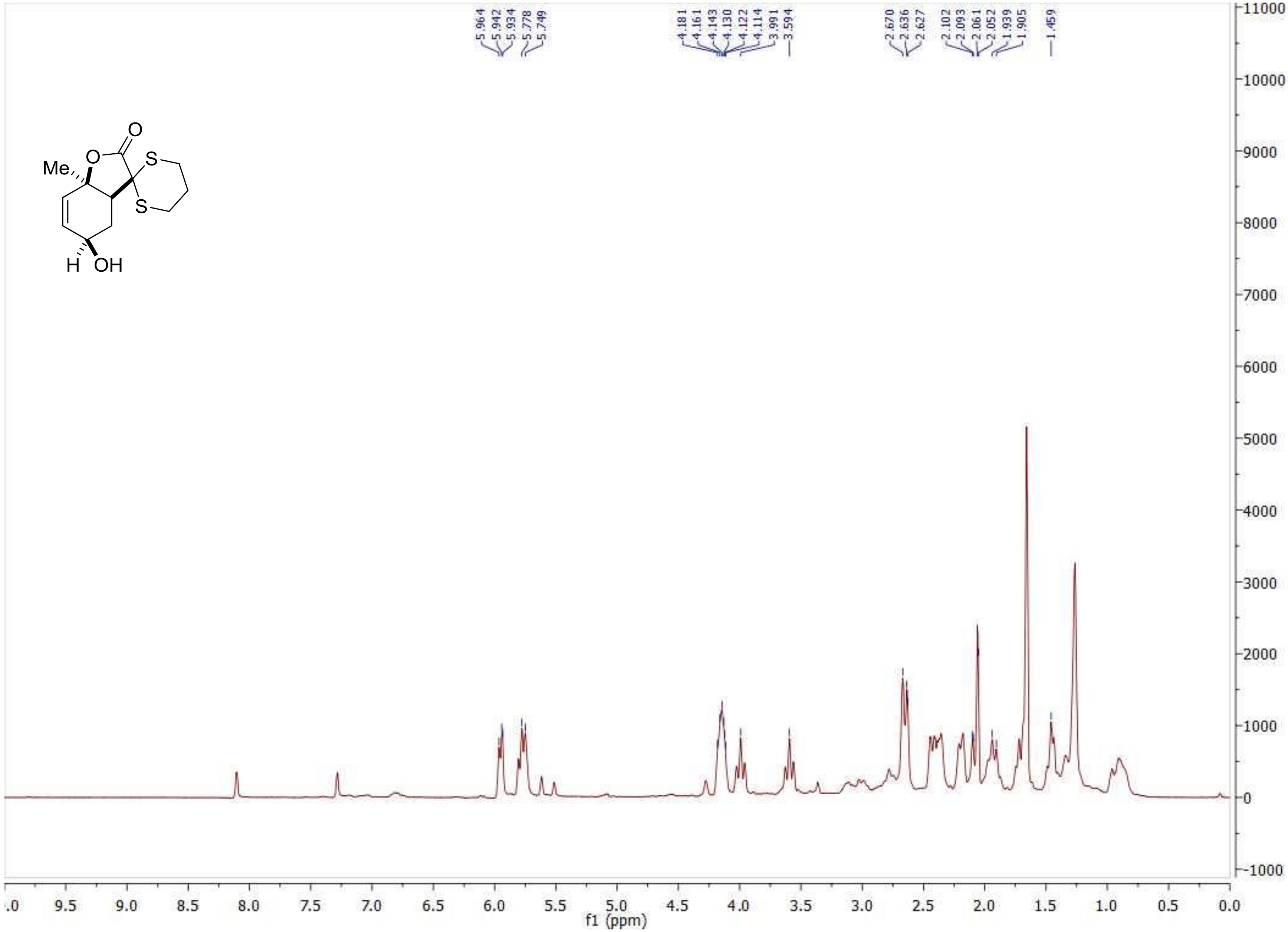

5.964
5.942
5.934
5.778
5.749
4.181
4.161
4.143
4.130
4.122
4.114
3.991
3.594
2.670
2.636
2.627
2.102
2.093
2.061
2.052
1.939
1.905
1.459
11000
10000
9000
8000
7000
6000
5000
4000
3000
2000
1000
0
-1000
9.5
9.0
8.5
8.0
7.5
7.0
6.5
6.0
5.5
5.0
4.5
4.0
3.5
3.0
2.5
2.0
1.5
1.0
0.5
0.0
f1 (ppm)

^{1}H and ^{13}C NMR spectra of new compounds:

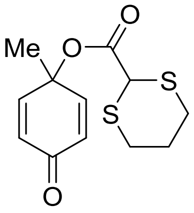

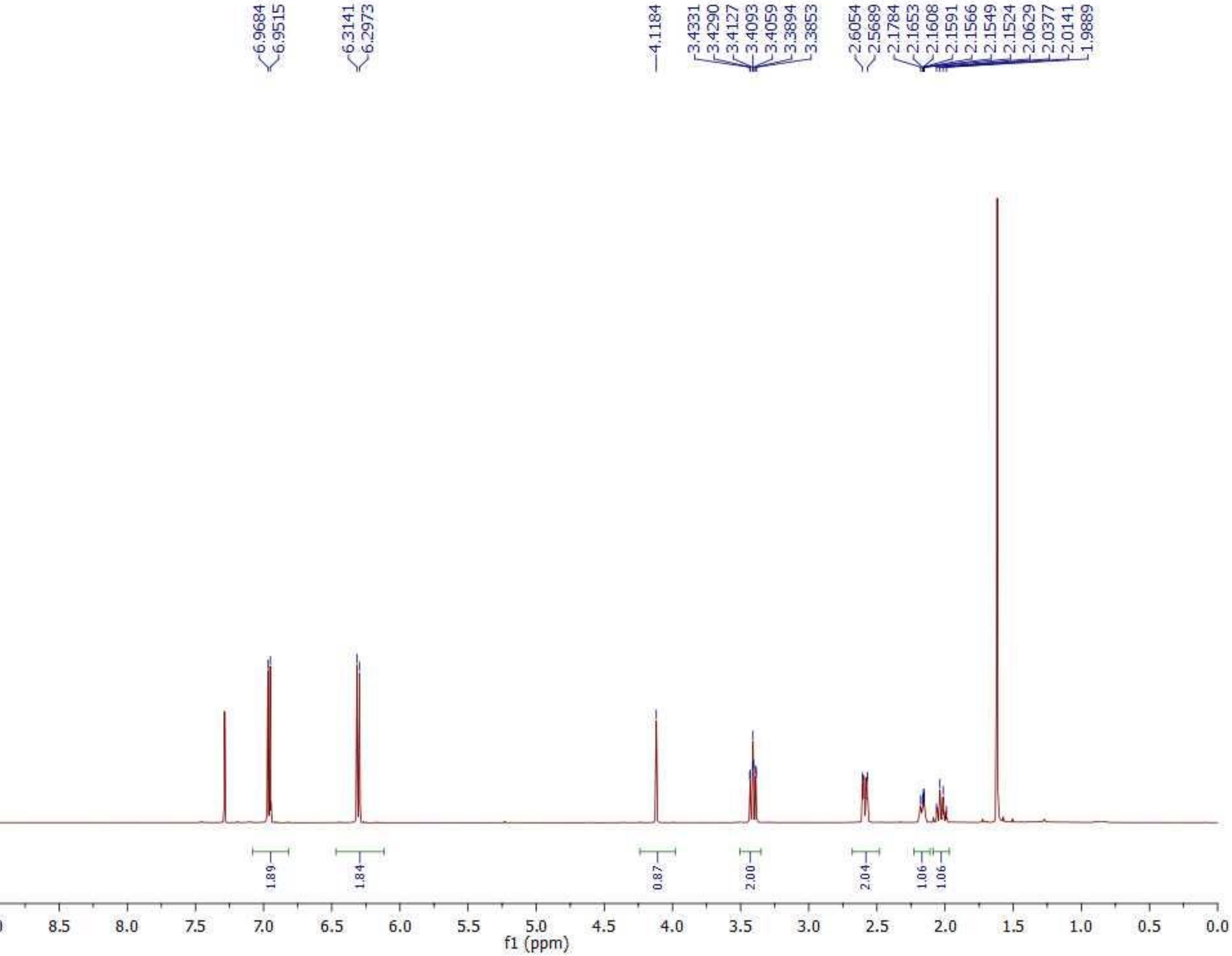

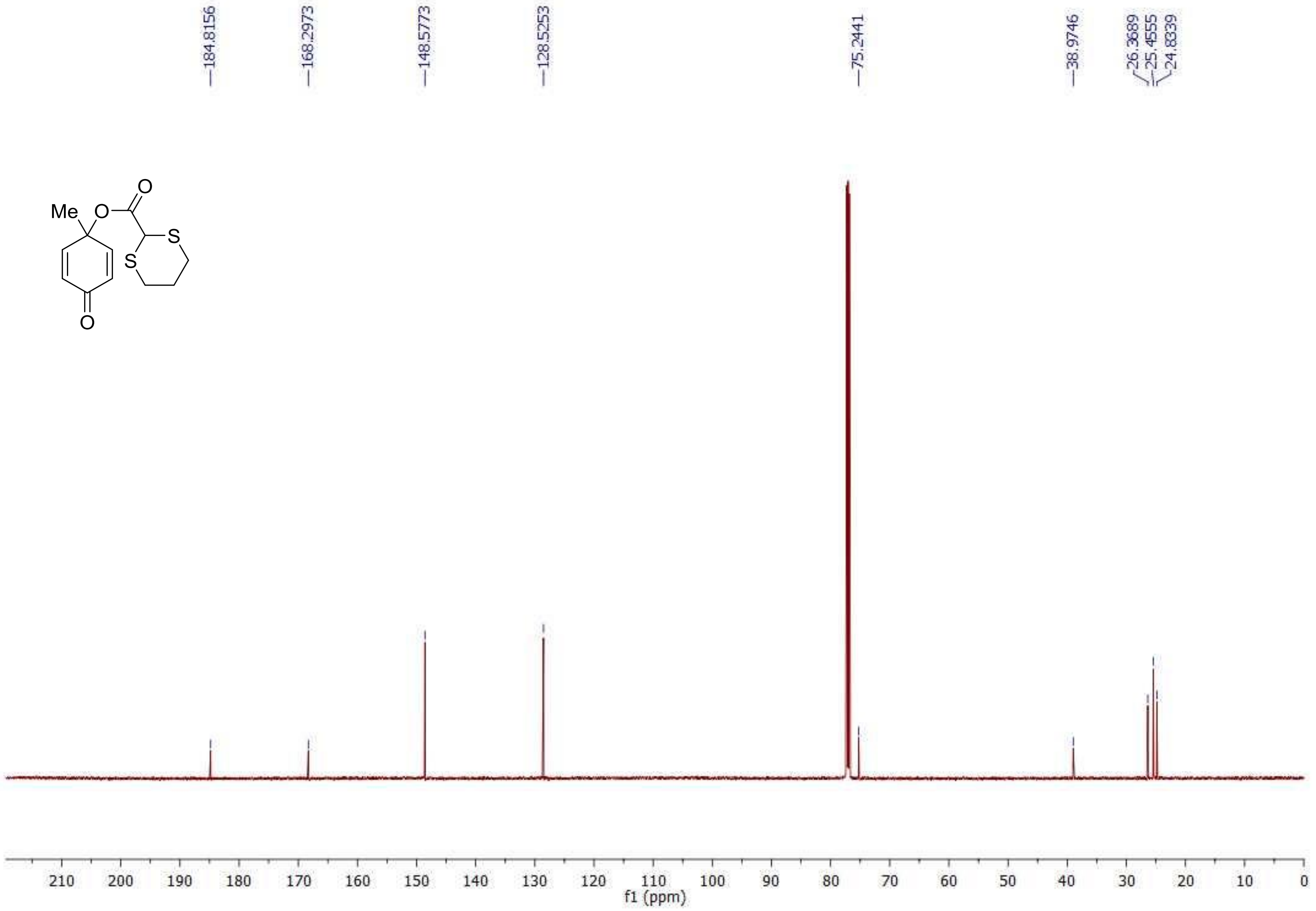
Me
O
O
S
S
O
184.8156
168.2973
148.5773
128.5253
75.2441
38.9746
26.3689
25.4555
24.8339
210
200
190
180
170
160
150
140
130
120
110
100
90
80
70
60
50
40
30
20
10
0
f1 (ppm)

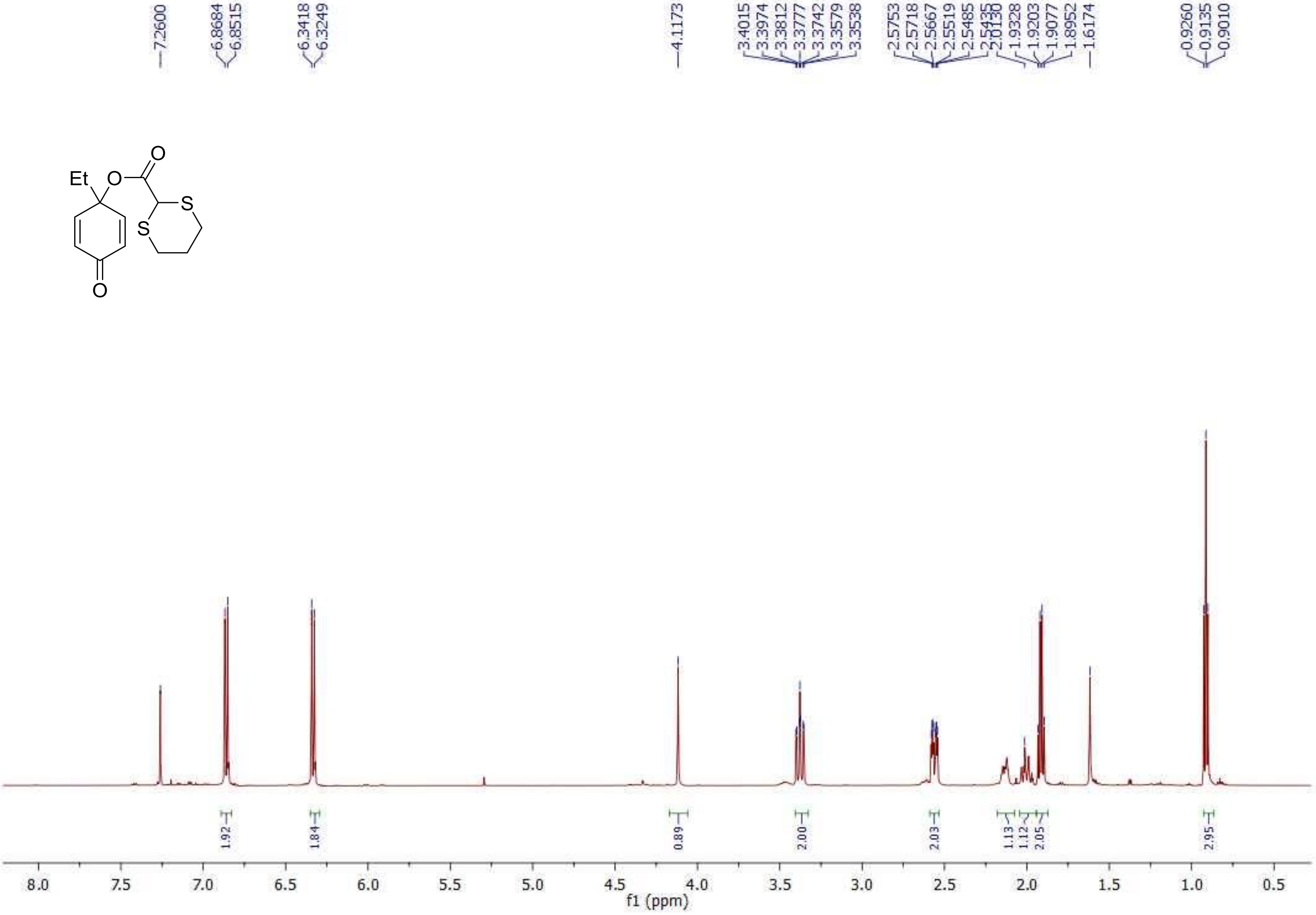
Et
O
O
S
S
O
7.2600
6.8684
6.8515
6.3418
6.3249
4.1173
3.4015
3.3974
3.3812
3.3777
3.3742
3.3579
3.3538
2.5753
2.5718
2.5667
2.5519
2.5485
2.5435
2.0130
1.9328
1.9203
1.9077
1.8952
1.6174
0.9260
0.9135
0.9010
1.92
1.84
0.89
2.00
2.03
1.13
1.12
2.05
2.95
8.0
7.5
7.0
6.5
6.0
5.5
5.0
4.5
4.0
3.5
3.0
2.5
2.0
1.5
1.0
0.5
f1 (ppm)

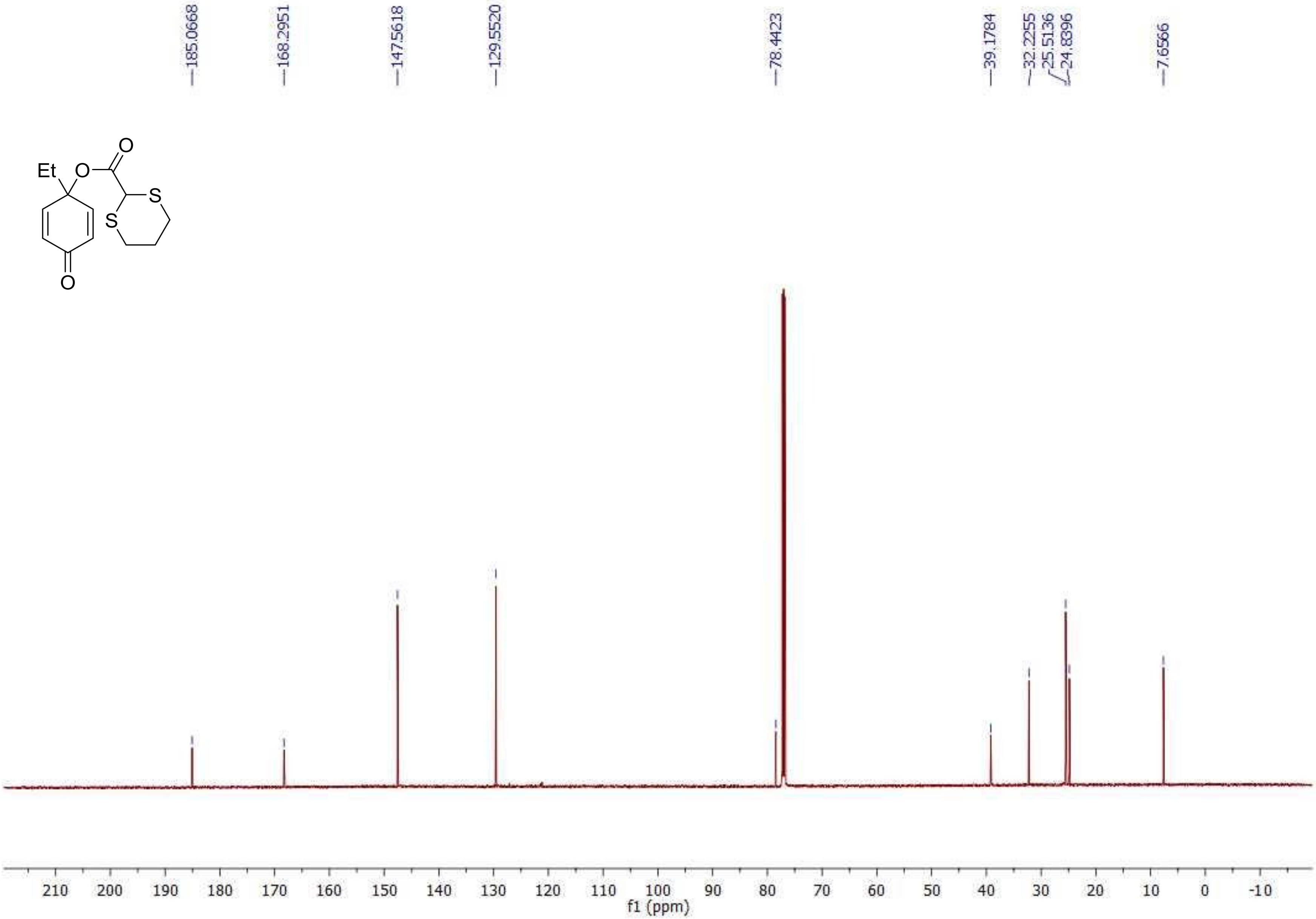

185.0668
168.2951
147.5618
129.5520
78.4423
39.1784
32.2255
25.5136
24.8396
7.6566
Et
O
O
S
S
O
210 200 190 180 170 160 150 140 130 120 110 100 90 80 70 60 50 40 30 20 10 0 -10
f1 (ppm)

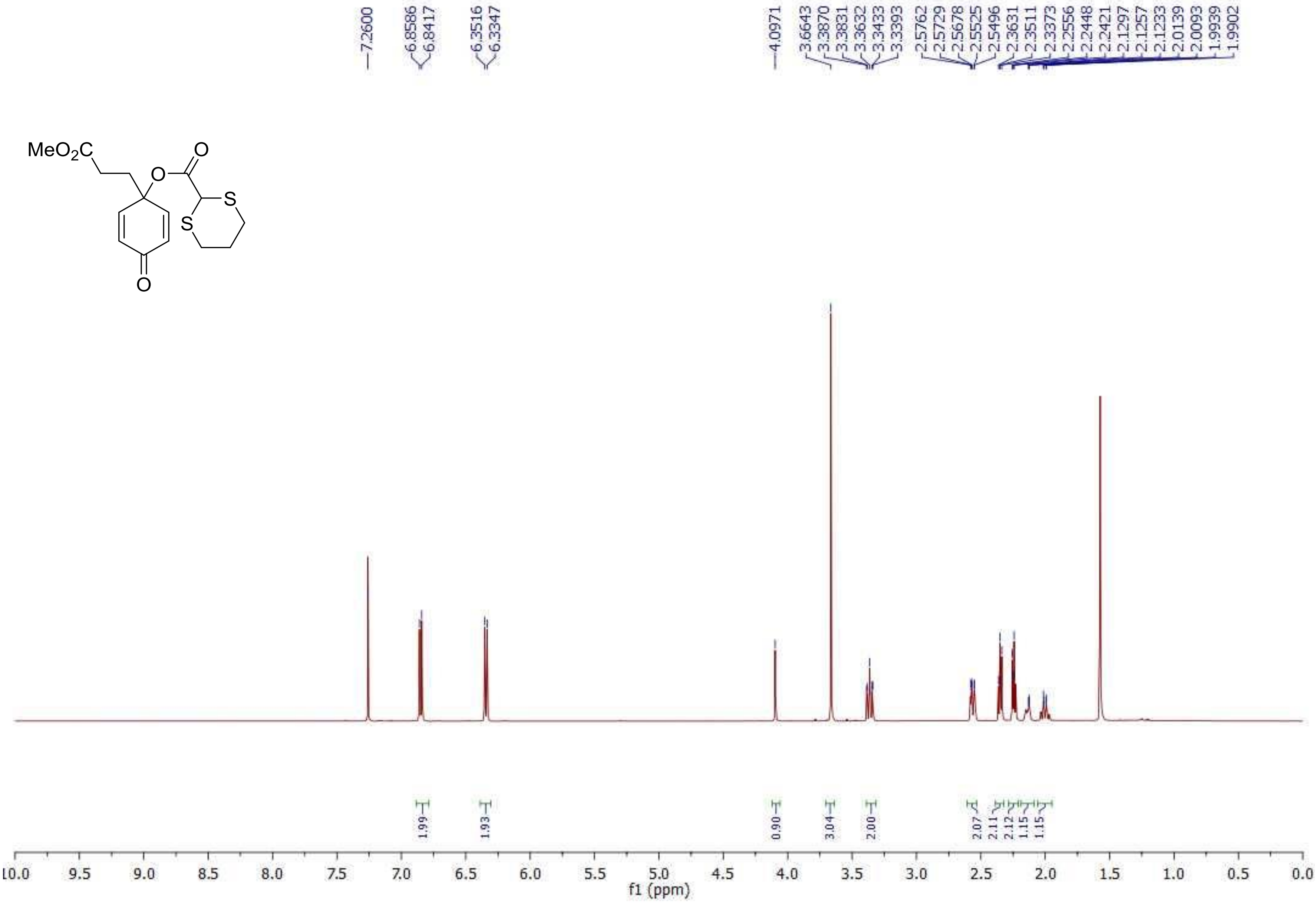

7.2600
6.8586
6.8417
6.3516
6.3347
4.0971
3.6643
3.3870
3.3831
3.3632
3.3433
3.3393
2.5762
2.5729
2.5678
2.5525
2.5496
2.3631
2.3511
2.3373
2.2556
2.2448
2.2421
2.1297
2.1257
2.1233
2.0139
2.0093
1.9939
1.9902
1.99
1.93
0.90
3.04
2.00
2.07
2.11
2.12
1.15
1.15
10.0 9.5 9.0 8.5 8.0 7.5 7.0 6.5 6.0 5.5 5.0 4.5 4.0 3.5 3.0 2.5 2.0 1.5 1.0 0.5 0.0
f1 (ppm)

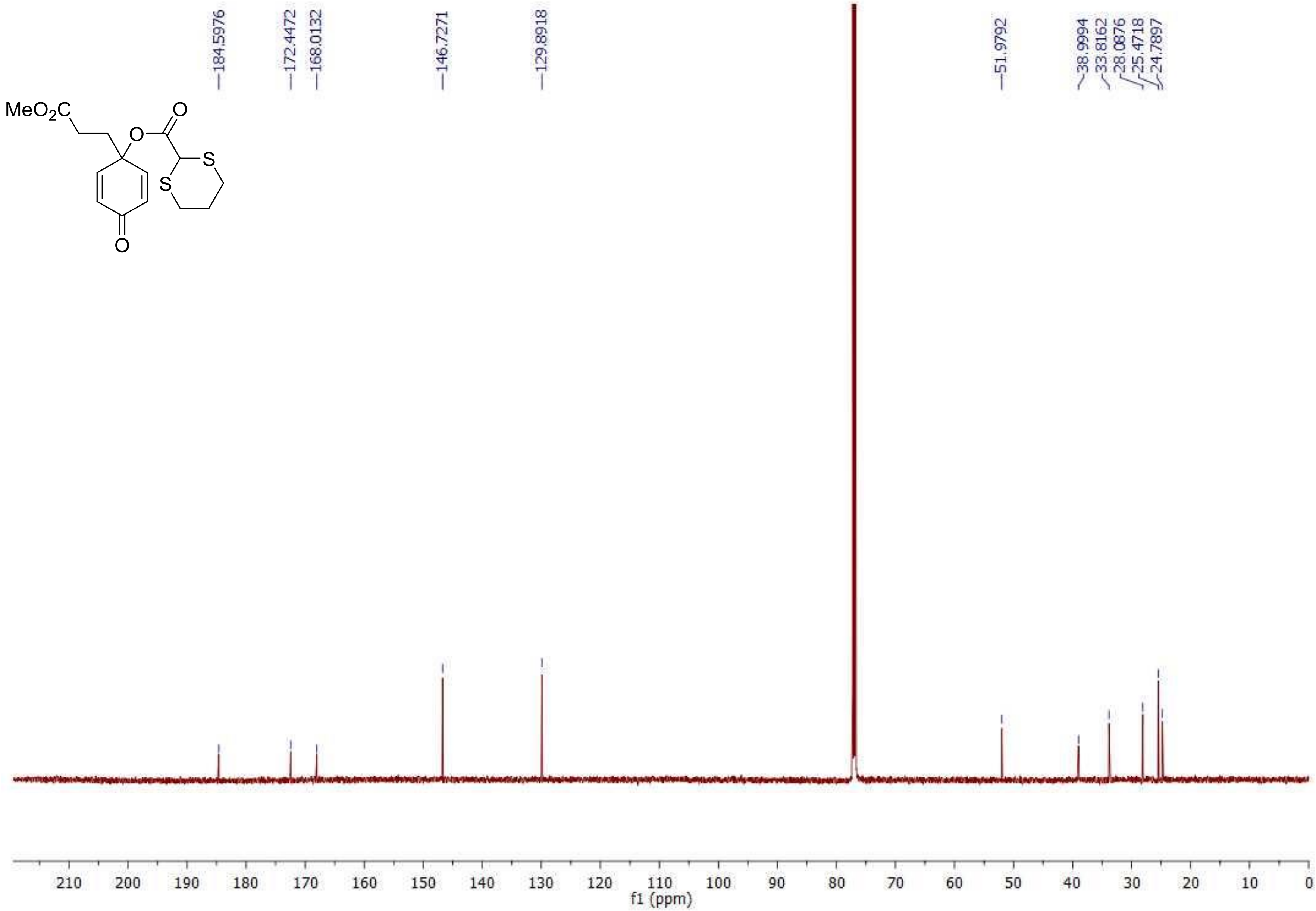
MeO2C
O
O
S
S
O
184.5976
172.4472
168.0132
146.7271
129.8918
51.9792
38.9994
33.8162
28.0876
25.4718
24.7897
210
200
190
180
170
160
150
140
130
120
110
100
90
80
70
60
50
40
30
20
10
0
f1 (ppm)

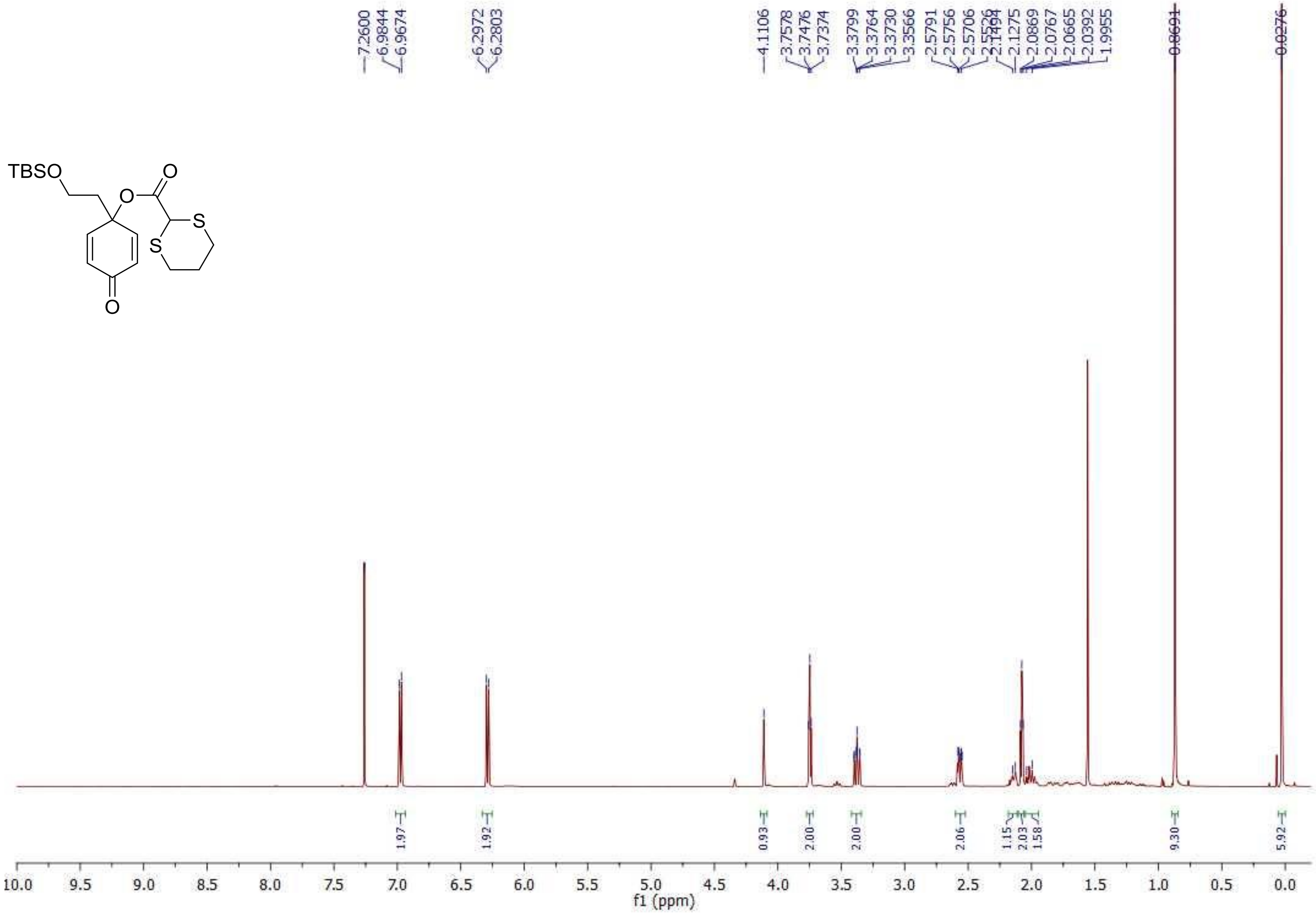
TBSO
O
O
S
S
O
7.2600
6.9844
6.9674
6.2972
6.2803
4.1106
3.7578
3.7476
3.7374
3.3799
3.3764
3.3730
3.3566
2.5791
2.5756
2.5706
2.5526
2.1494
2.1275
2.0869
2.0767
2.0665
2.0392
1.9955
0.8691
0.0276
1.97
1.92
0.93
2.00
2.00
2.06
1.15
2.03
1.58
9.30
5.92
10.0 9.5 9.0 8.5 8.0 7.5 7.0 6.5 6.0 5.5 5.0 4.5 4.0 3.5 3.0 2.5 2.0 1.5 1.0 0.5 0.0
f1 (ppm)

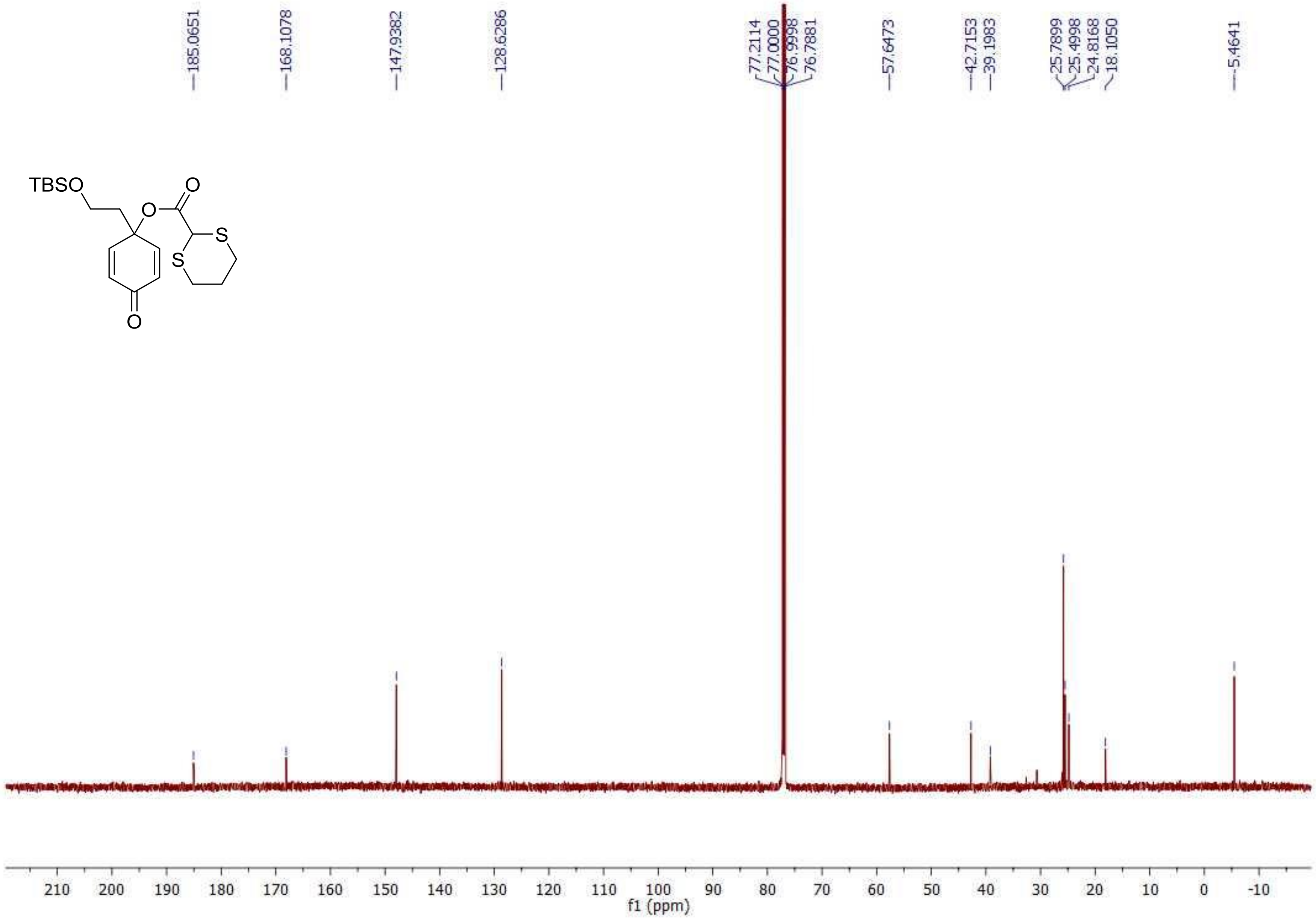
185.0651
168.1078
147.9382
128.6286
77.2114
77.0000
76.9998
76.7881
57.6473
42.7153
39.1983
25.7899
25.4998
24.8168
18.1050
-5.4641
TBSO
O
O
S
S
O
210 200 190 180 170 160 150 140 130 120 110 100 90 80 70 60 50 40 30 20 10 0 -10
f1 (ppm)

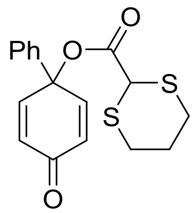

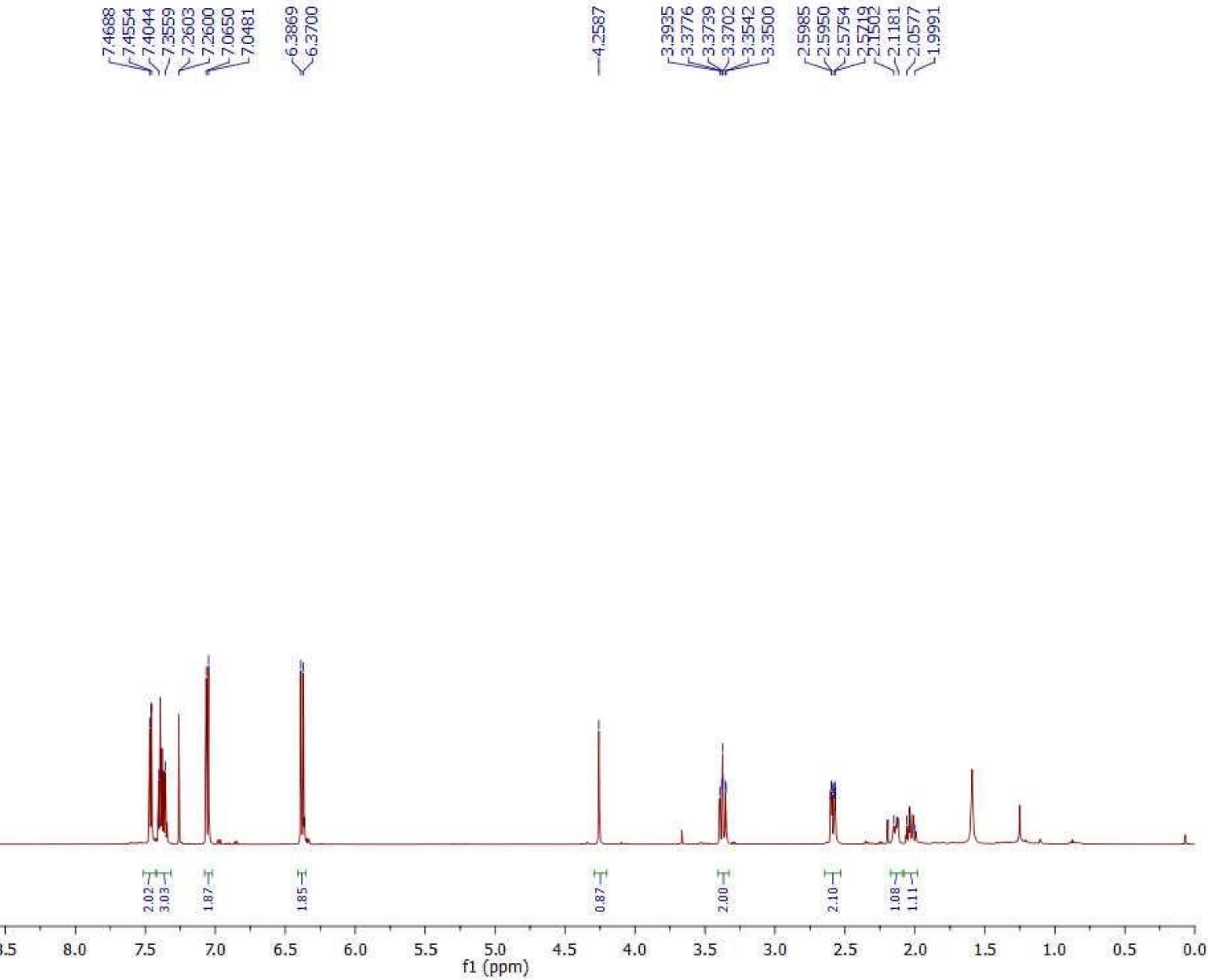
7.4688
7.4554
7.4044
7.3559
7.2603
7.2600
7.0650
7.0481
6.3869
6.3700
4.2587
3.3935
3.3776
3.3739
3.3702
3.3542
3.3500
2.5985
2.5950
2.5754
2.5719
2.1502
2.1181
2.0577
1.9991
2.02
3.03
1.87
1.85
0.87
2.00
2.10
1.08
1.11
f1 (ppm)

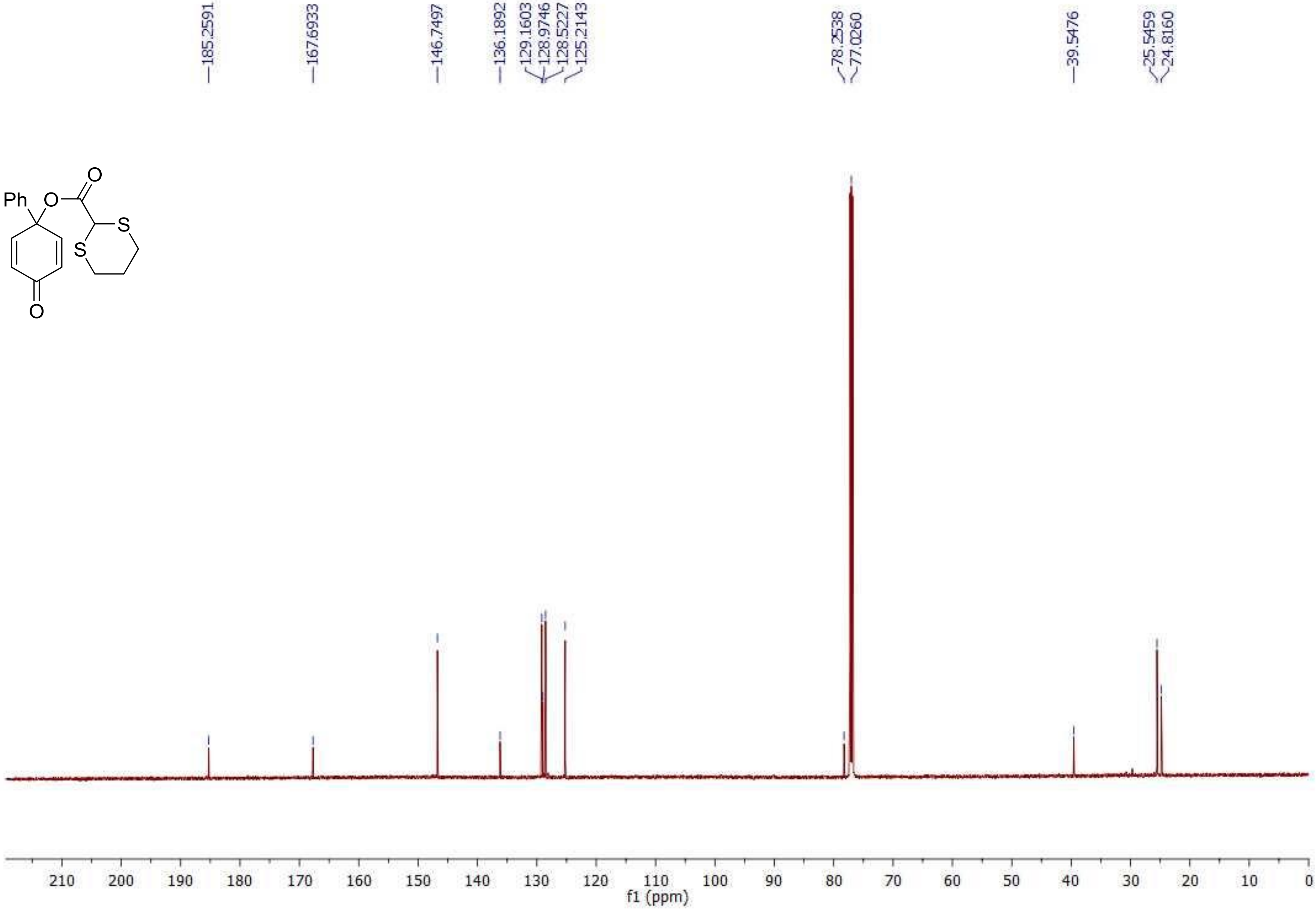
Ph
O
O
S
S
O
185.2591
167.6933
146.7497
136.1892
129.1603
128.9746
128.5227
125.2143
78.2538
77.0260
39.5476
25.5459
24.8160
210
200
190
180
170
160
150
140
130
120
110
100
90
80
70
60
50
40
30
20
10
0
f1 (ppm)

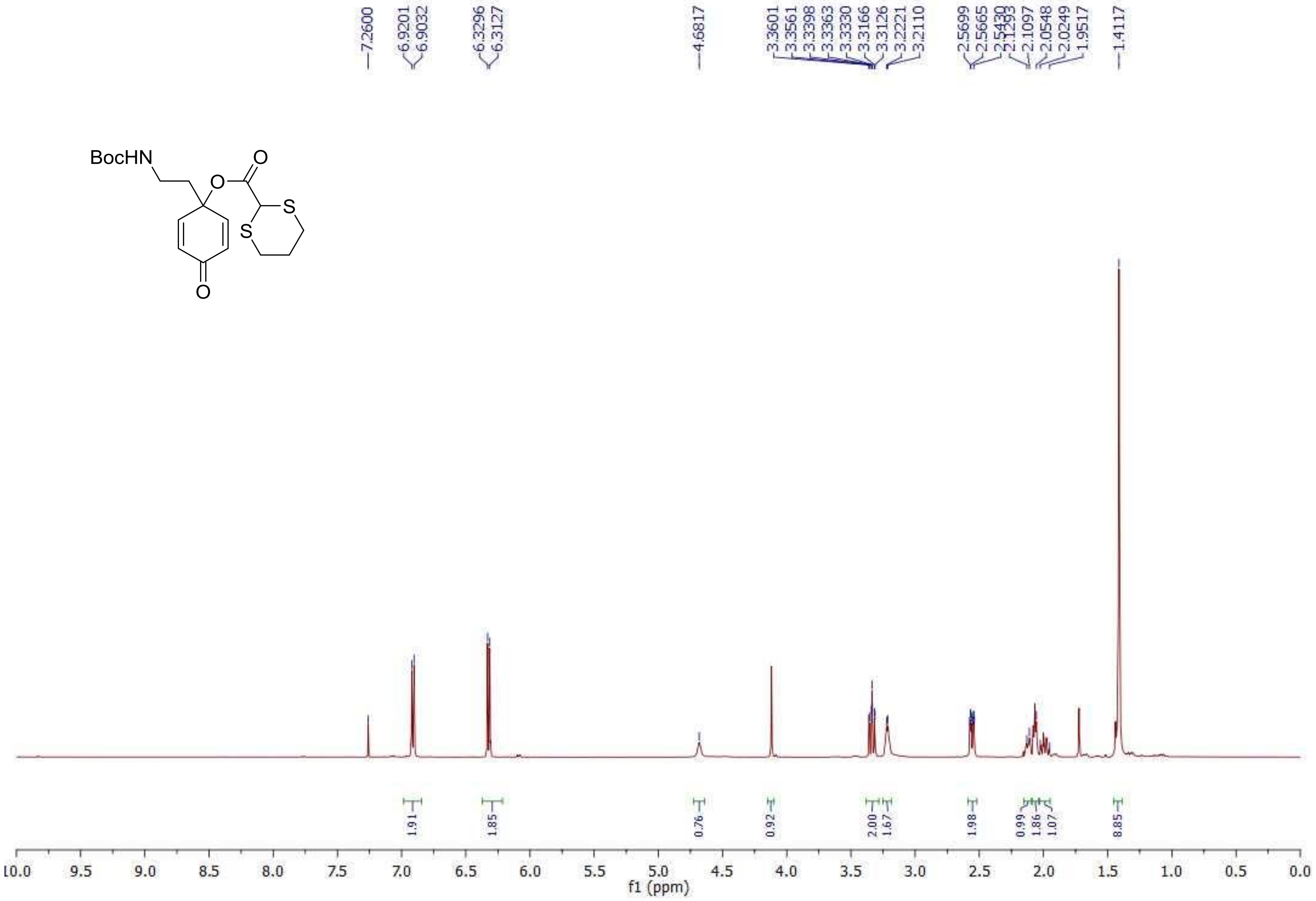
BocHN
O
O
S
S
O
7.2600
6.9201
6.9032
6.3296
6.3127
4.6817
3.3601
3.3561
3.3398
3.3363
3.3330
3.3166
3.3126
3.2221
3.2110
2.5699
2.5665
2.5430
2.1293
2.1097
2.0548
2.0249
1.9517
1.4117
1.91
1.85
0.76
0.92
2.00
1.67
1.98
0.99
1.86
1.07
8.85
10.0
9.5
9.0
8.5
8.0
7.5
7.0
6.5
6.0
5.5
5.0
4.5
4.0
3.5
3.0
2.5
2.0
1.5
1.0
0.5
0.0
f1 (ppm)

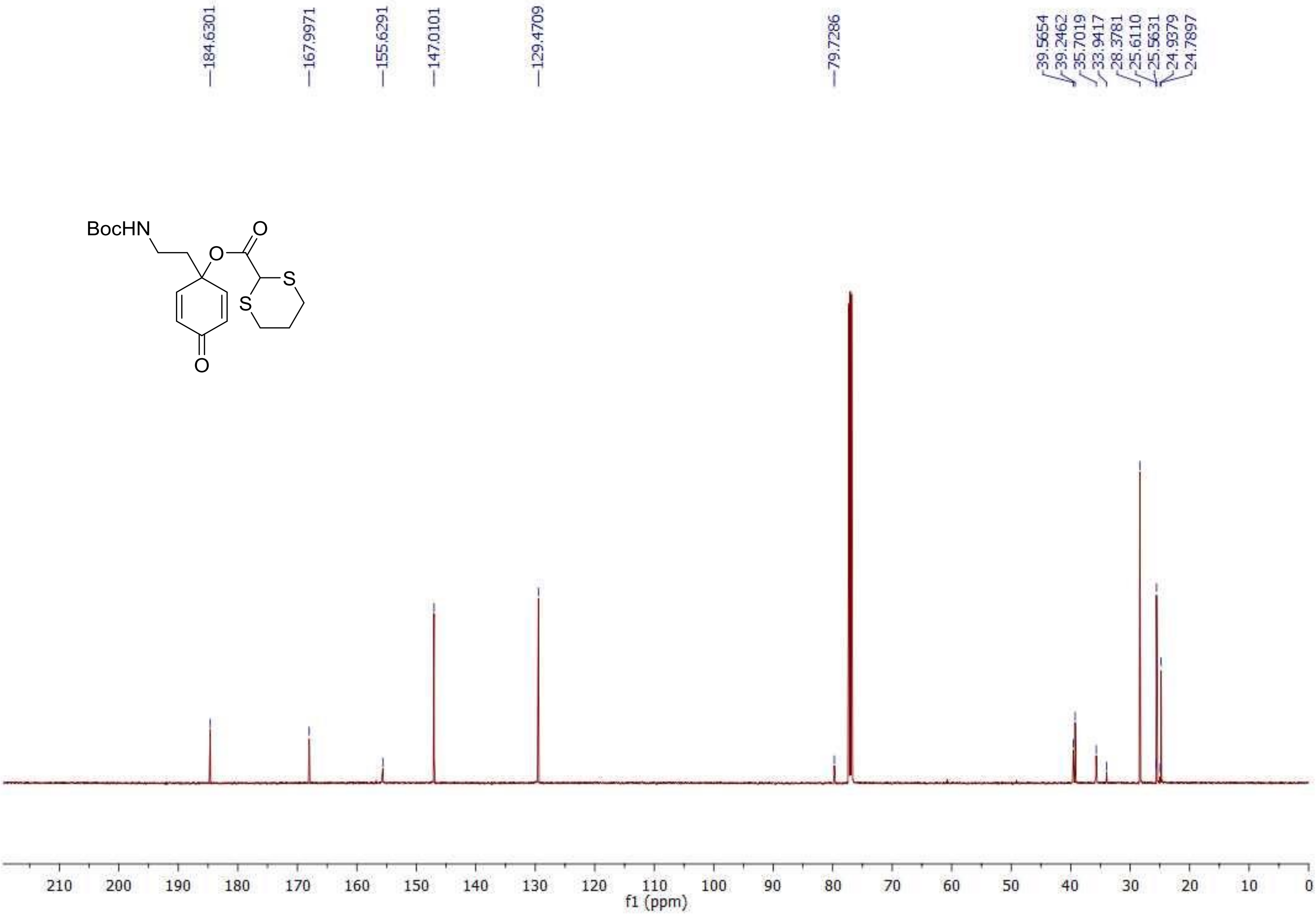
184.6301
167.9971
155.6291
147.0101
129.4709
79.7286
39.5654
39.2462
35.7019
33.9417
28.3781
25.6110
25.5631
24.9379
24.7897
BocHN
O
O
S
S
O
210 200 190 180 170 160 150 140 130 120 110 100 90 80 70 60 50 40 30 20 10 0
f1 (ppm)

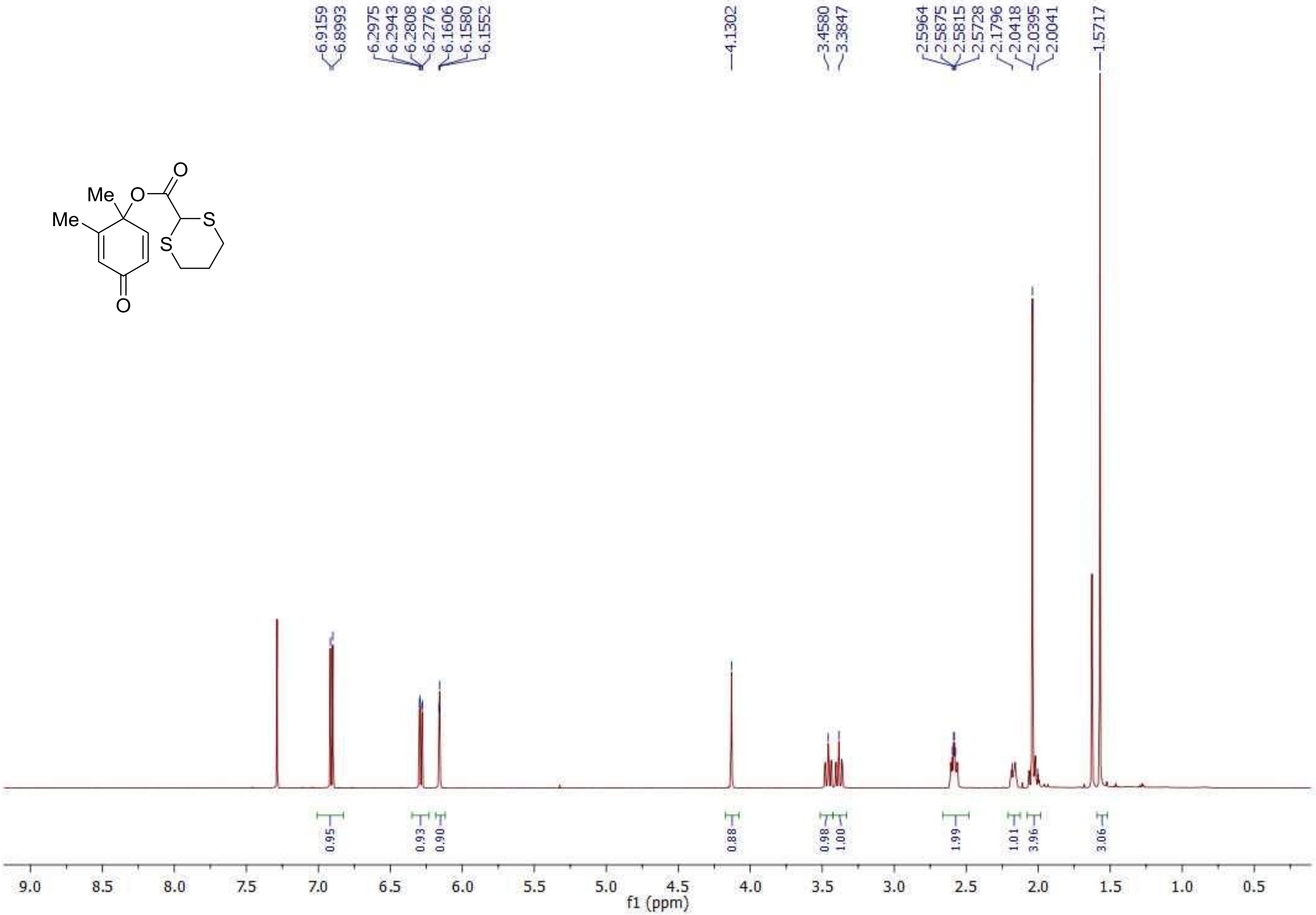
Me
Me
O
O
S
S
O
6.9159
6.8993
6.2975
6.2943
6.2808
6.2776
6.1606
6.1580
6.1552
4.1302
3.4580
3.3847
2.5964
2.5875
2.5815
2.5728
2.1796
2.0418
2.0395
2.0041
1.5717
0.95
0.93
0.90
0.88
0.98
1.00
1.99
1.01
3.96
3.06
9.0
8.5
8.0
7.5
7.0
6.5
6.0
5.5
5.0
4.5
4.0
3.5
3.0
2.5
2.0
1.5
1.0
0.5
f1 (ppm)

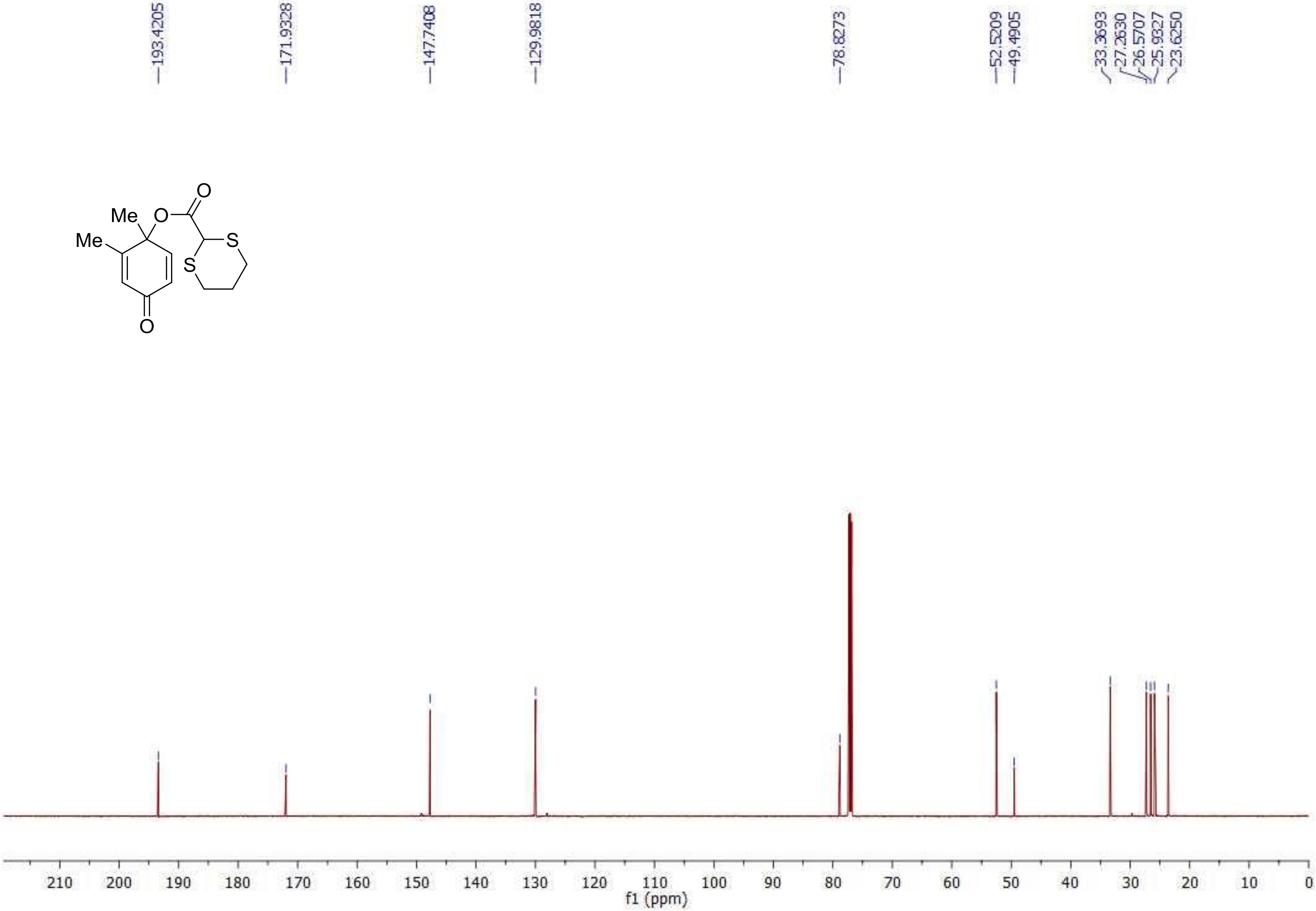
193.4205
171.9328
147.7408
129.9818
78.8273
52.5209
49.4905
33.3693
27.2630
26.5707
25.9327
23.6250
Me
Me
O
O
O
S
S
210 200 190 180 170 160 150 140 130 120 110 100 90 80 70 60 50 40 30 20 10 0
f1 (ppm)

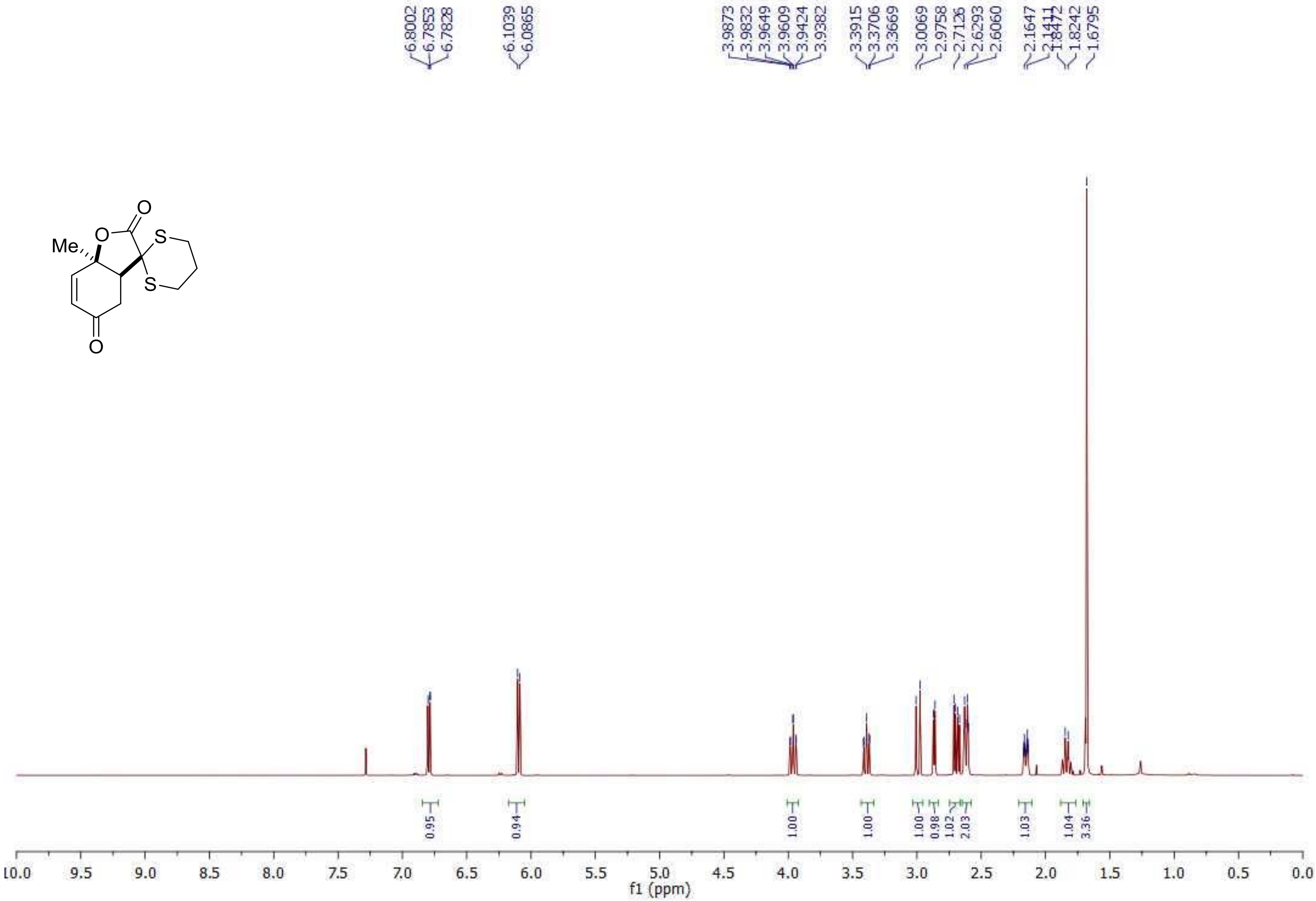
6.8002
6.7853
6.7828
6.1039
6.0865
3.9873
3.9832
3.9649
3.9609
3.9424
3.9382
3.3915
3.3706
3.3669
3.0069
2.9758
2.7126
2.6293
2.6060
2.1647
2.1411
1.8472
1.8242
1.6795
0.95
0.94
1.00
1.00
1.00
0.98
1.02
2.03
1.03
1.04
3.36
10.0 9.5 9.0 8.5 8.0 7.5 7.0 6.5 6.0 5.5 5.0 4.5 4.0 3.5 3.0 2.5 2.0 1.5 1.0 0.5 0.0
f1 (ppm)

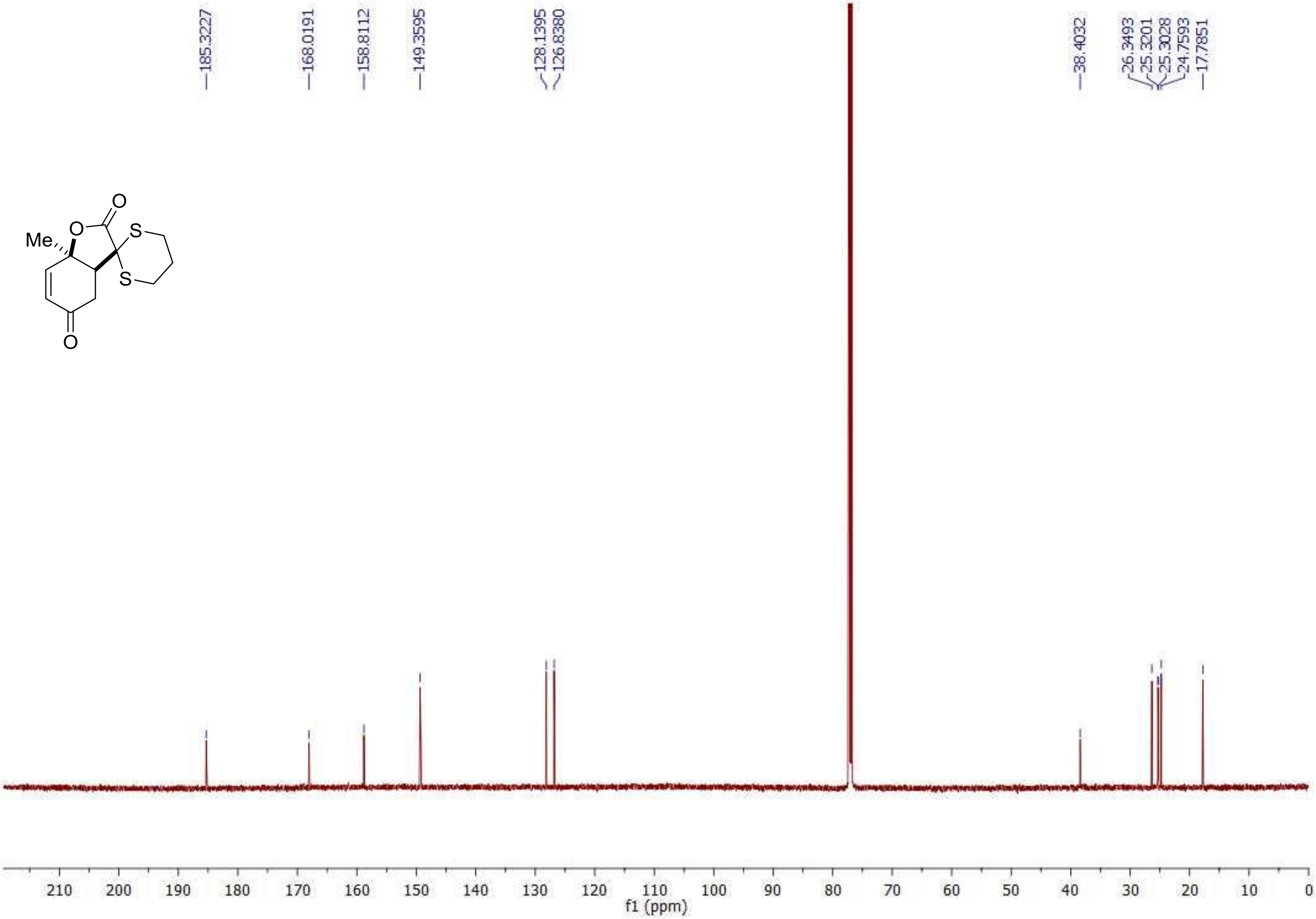
185.3227
168.0191
158.8112
149.3595
128.1395
126.8380
38.4032
26.3493
25.3201
25.3028
24.7593
17.7851
Me
O
O
S
S
O
210
200
190
180
170
160
150
140
130
120
110
100
90
80
70
60
50
40
30
20
10
0
f1 (ppm)

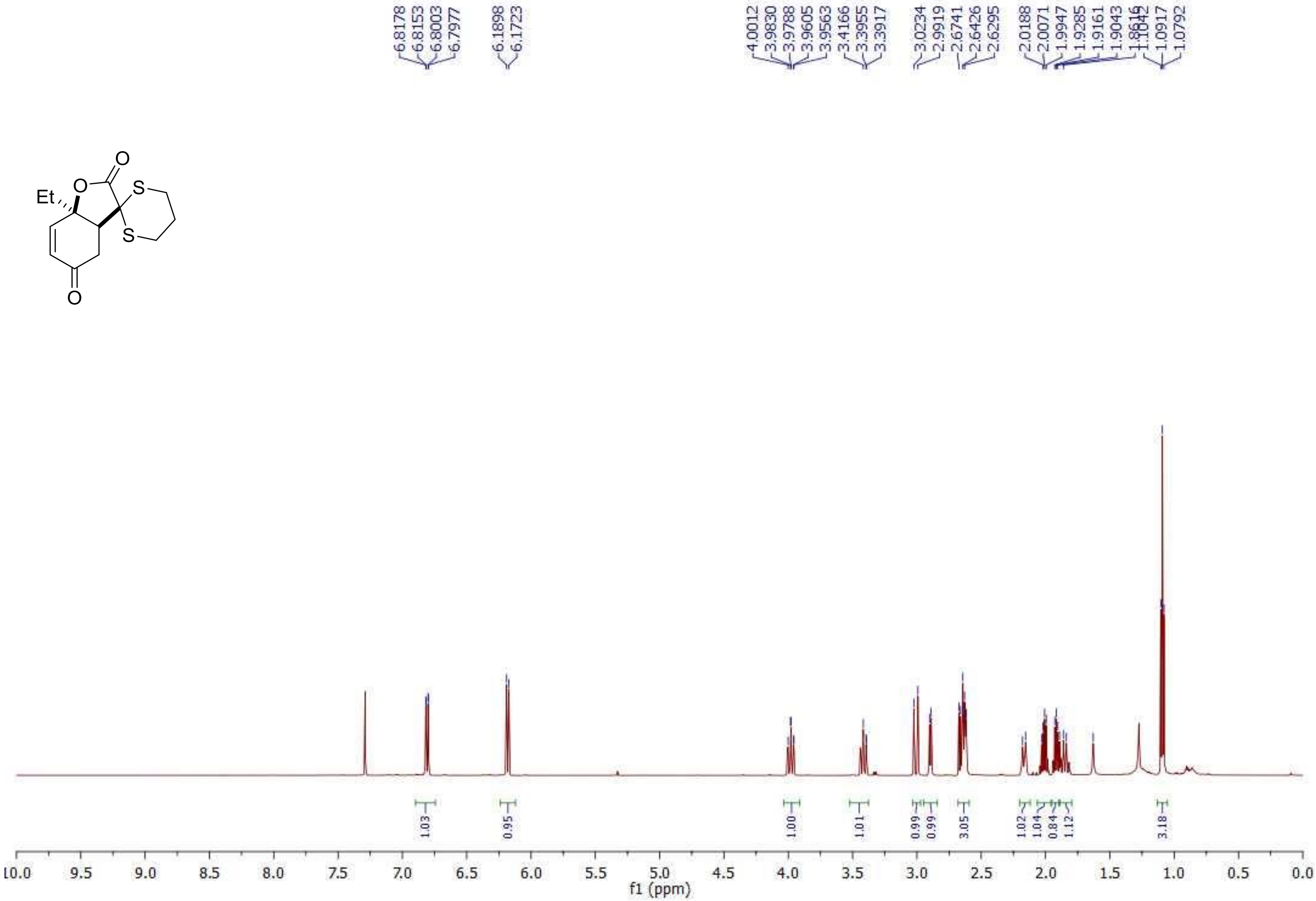

Et
O
O
S
S
O
6.8178
6.8153
6.8003
6.7977
6.1898
6.1723
4.0012
3.9830
3.9788
3.9605
3.9563
3.4166
3.3955
3.3917
3.0234
2.9919
2.6741
2.6426
2.6295
2.0188
2.0071
1.9947
1.9285
1.9161
1.9043
1.8616
1.1042
1.0917
1.0792
1.03
0.95
1.00
1.01
0.99
0.99
3.05
1.02
1.04
0.84
1.12
3.18
f1 (ppm)

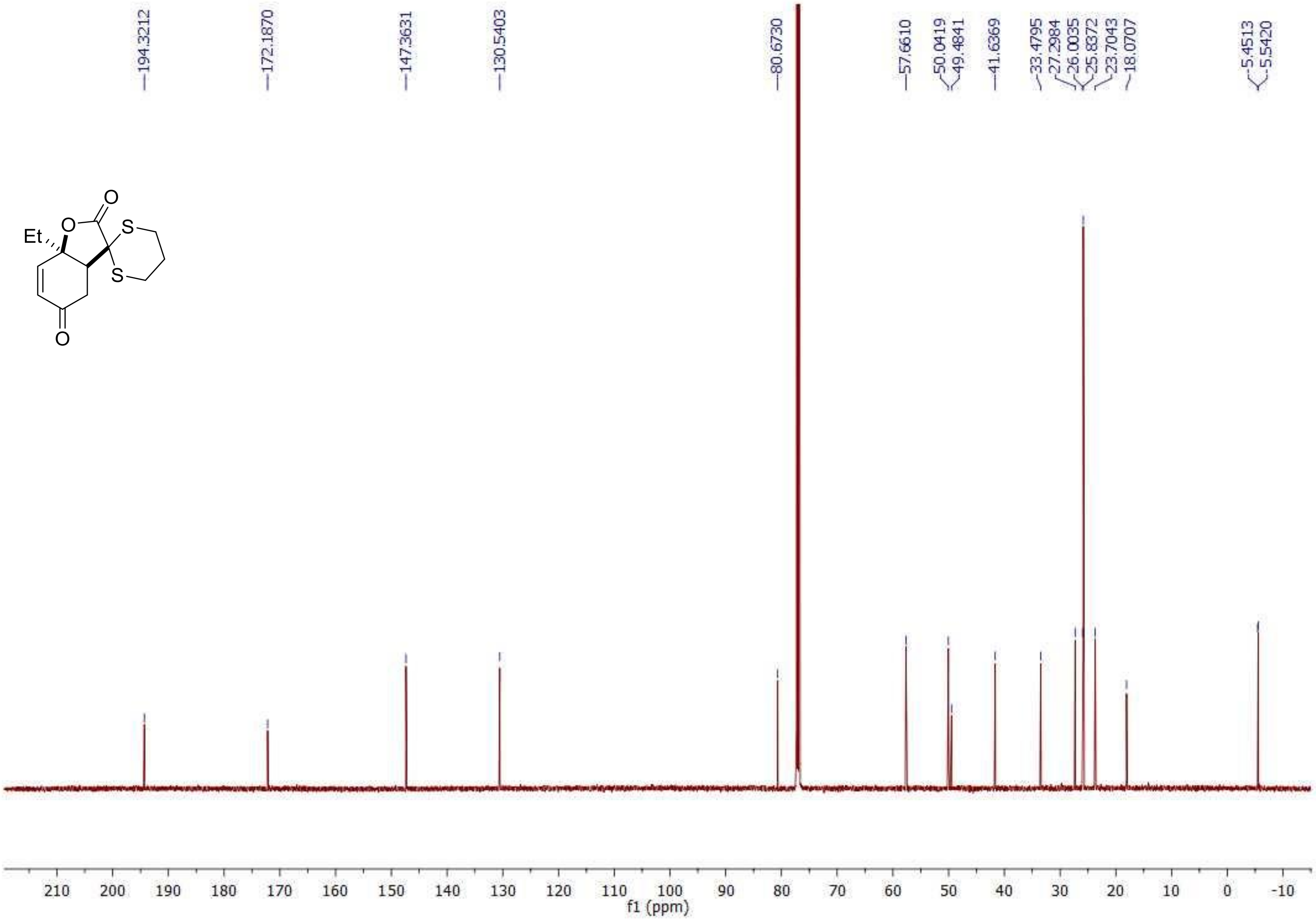
194.3212
172.1870
147.3631
130.5403
80.6730
57.6610
50.0419
49.4841
41.6369
33.4795
27.2984
26.0035
25.8372
23.7043
18.0707
-5.4513
-5.5420
Et
O
O
S
S
O
210 200 190 180 170 160 150 140 130 120 110 100 90 80 70 60 50 40 30 20 10 0 -10
f1 (ppm)

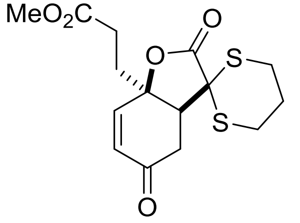
MeO2C
O
O
S
S
O

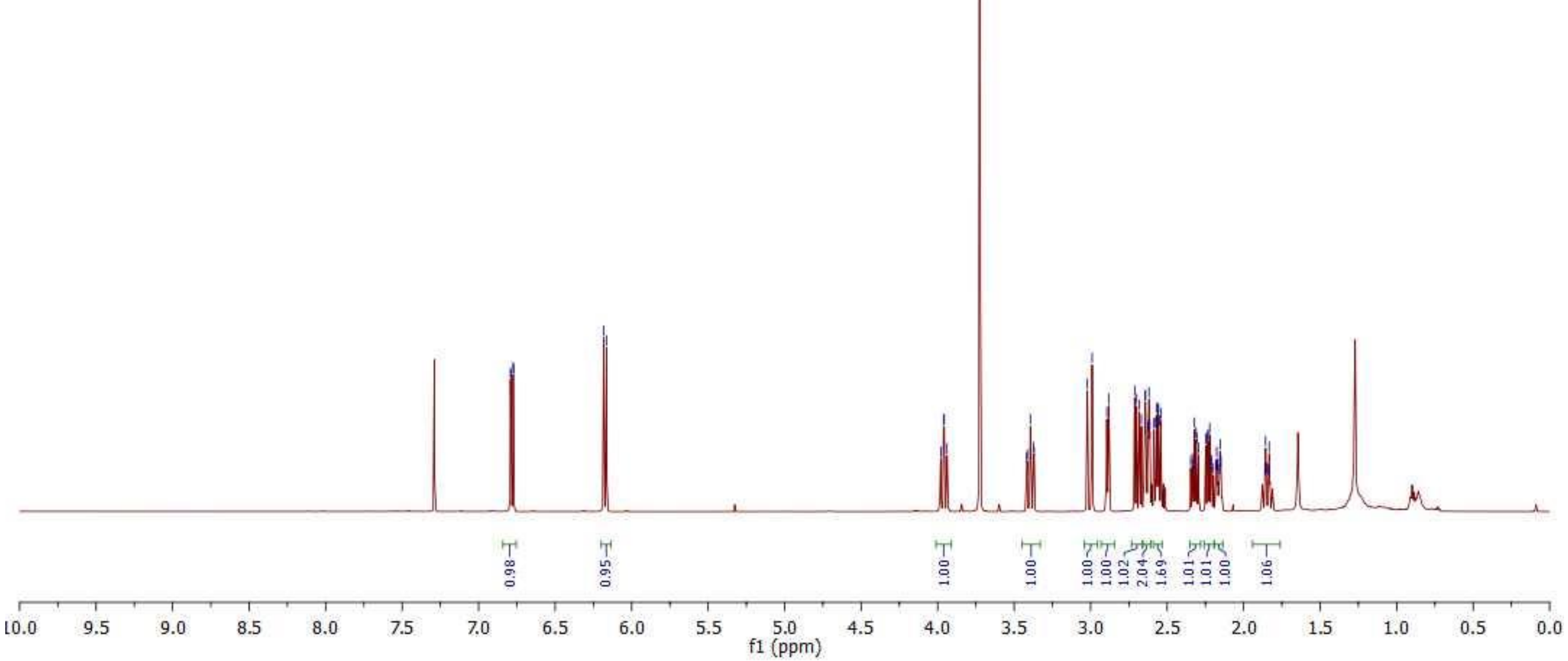
6.7918
6.7891
6.7742
6.7716
6.1820
6.1644
3.9793
3.9612
3.9571
3.9386
3.3938
3.3726
3.3688
3.0217
2.9902
2.8813
2.7125
2.6995
2.6429
2.6415
1.8564
1.8515
1.8381
1.8332
0.98
0.95
1.00
1.00
1.00
1.00
1.02
2.04
1.69
1.01
1.01
1.00
1.06
10.0 9.5 9.0 8.5 8.0 7.5 7.0 6.5 6.0 5.5 5.0 4.5 4.0 3.5 3.0 2.5 2.0 1.5 1.0 0.5 0.0
f1 (ppm)

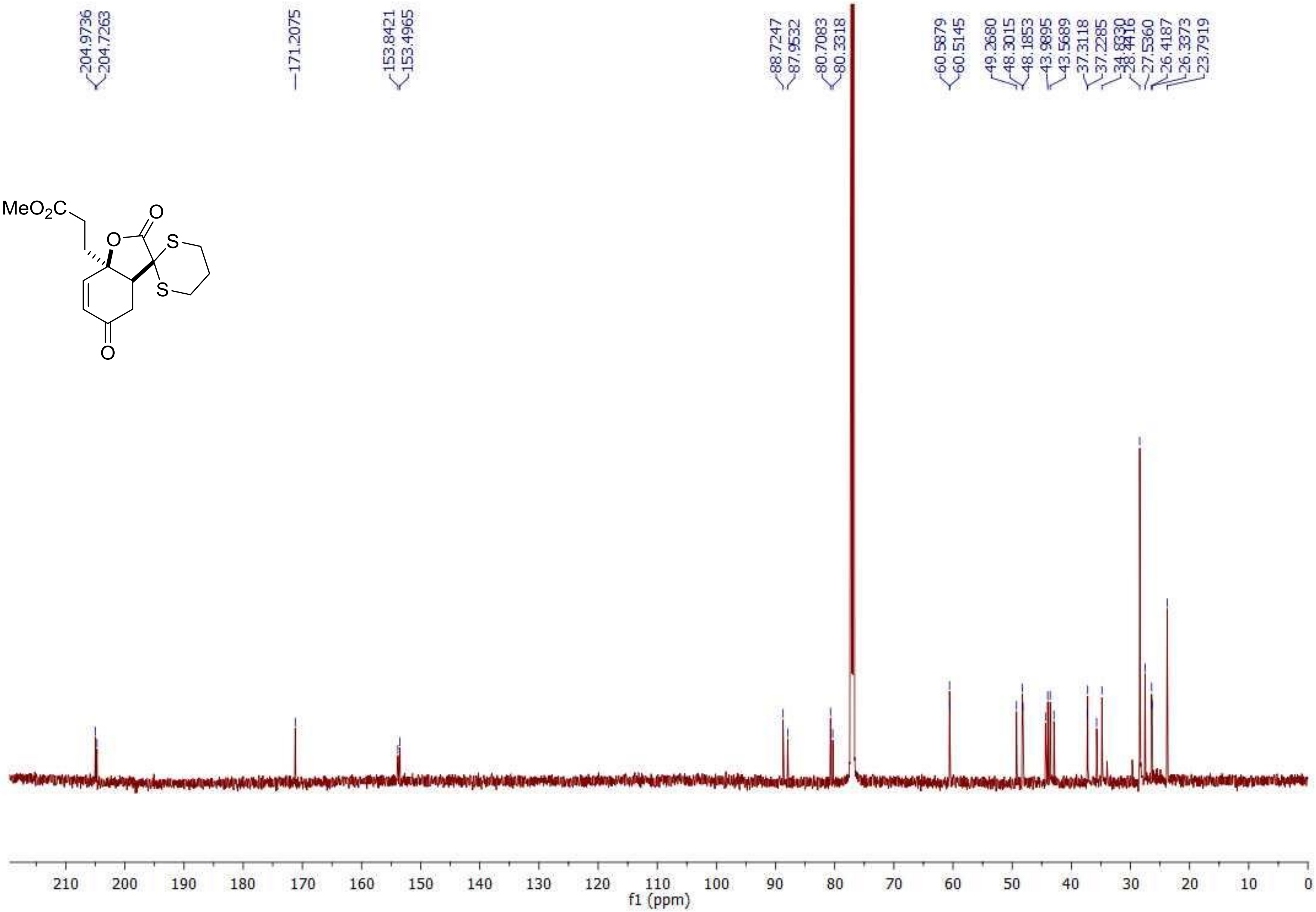
204.9736
204.7263
171.2075
153.8421
153.4965
88.7247
87.9532
80.7083
80.3318
60.5879
60.5145
49.2680
48.3015
48.1853
43.9895
43.5689
37.3118
37.2285
27.5360
26.4187
26.3373
23.7919
MeO2C
O
S
f1 (ppm)

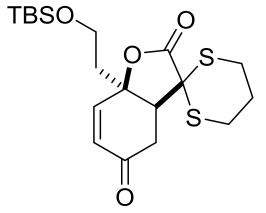

TBSO
O
O
S
S
O

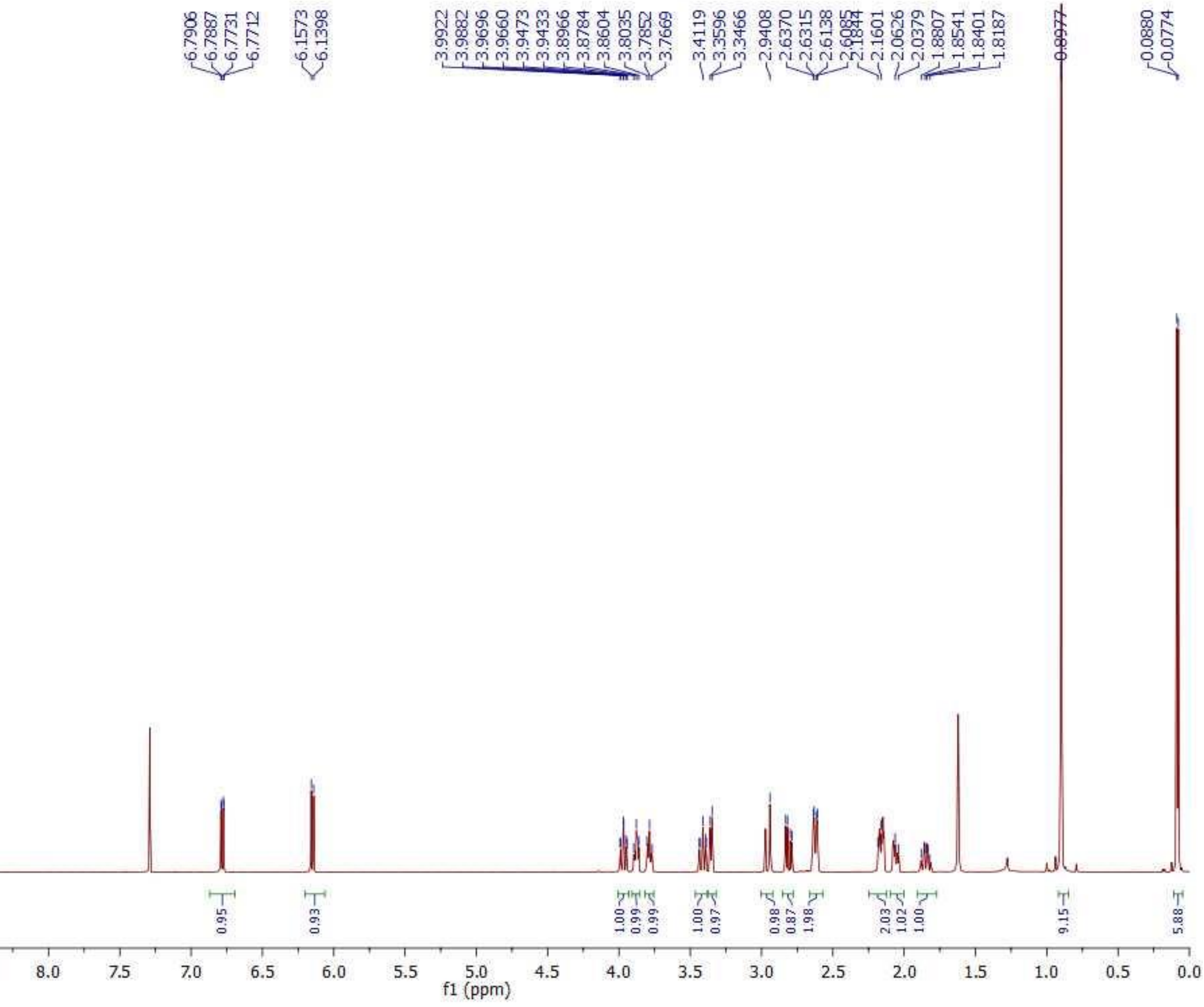

6.7906
6.7887
6.7731
6.7712
6.1573
6.1398
3.9922
3.9882
3.9696
3.9660
3.9473
3.9433
3.8966
3.8784
3.8604
3.8035
3.7852
3.7669
3.4119
3.3596
3.3466
2.9408
2.6370
2.6315
2.6138
2.1601
2.0626
2.0379
1.8807
1.8541
1.8401
1.8187
0.0880
0.0774
0.95
0.93
1.00
0.99
0.99
1.00
0.97
0.98
0.87
1.98
2.03
1.02
1.00
9.15
5.88
10.0
9.5
9.0
8.5
8.0
7.5
7.0
6.5
6.0
5.5
5.0
4.5
4.0
3.5
3.0
2.5
2.0
1.5
1.0
0.5
0.0
f1 (ppm)

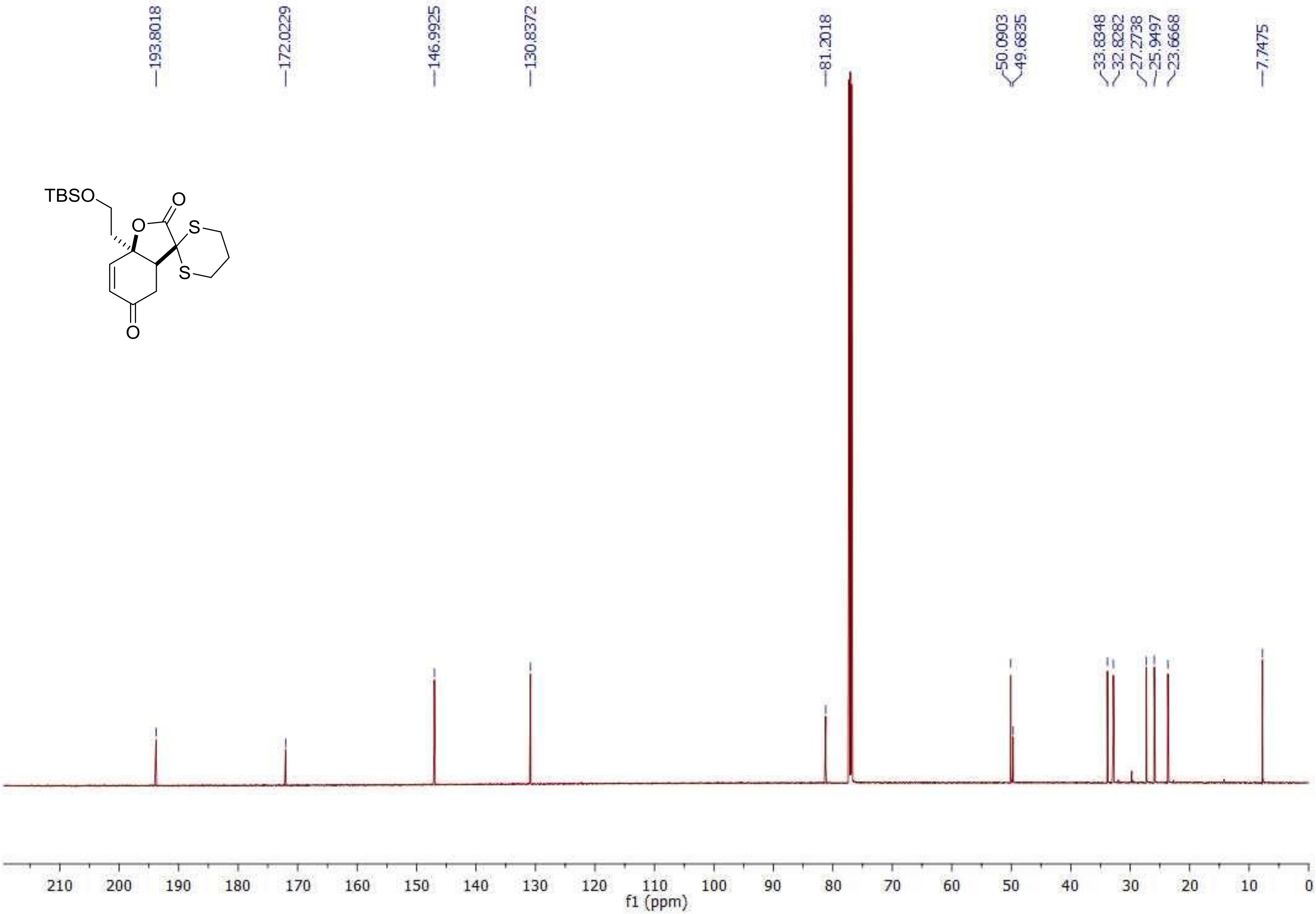

193.8018
172.0229
146.9925
130.8372
81.2018
50.0903
49.6835
33.8348
32.8282
27.2738
25.9497
23.6668
7.7475
TBSO
O
O
S
S
O
210
200
190
180
170
160
150
140
130
120
110
100
90
80
70
60
50
40
30
20
10
0
f1 (ppm)

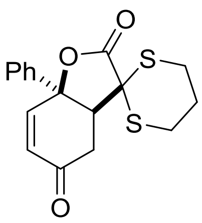
Ph
O
O
S
S
O

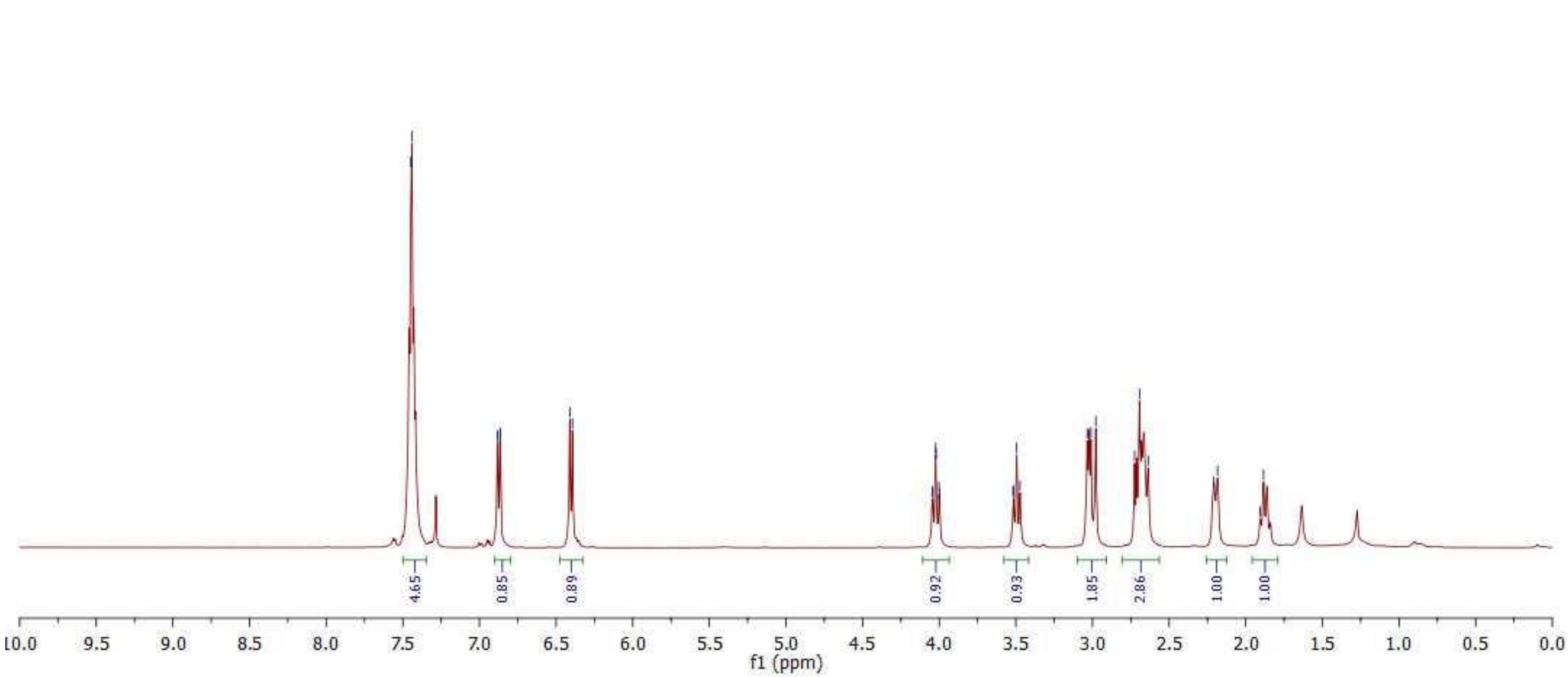
7.4462
7.4434
7.4402
6.8828
6.8808
6.8655
6.8635
6.4103
6.3930
4.0459
4.0423
4.0236
4.0204
4.0015
3.9980
3.5151
3.4946
3.4732
3.0346
3.0099
2.9783
2.7248
2.6934
2.6365
2.2102
2.1838
1.9062
1.8843
4.65
0.85
0.89
0.92
0.93
1.85
2.86
1.00
1.00
10.0
9.5
9.0
8.5
8.0
7.5
7.0
6.5
6.0
5.5
5.0
4.5
4.0
3.5
3.0
2.5
2.0
1.5
1.0
0.5
0.0
f1 (ppm)

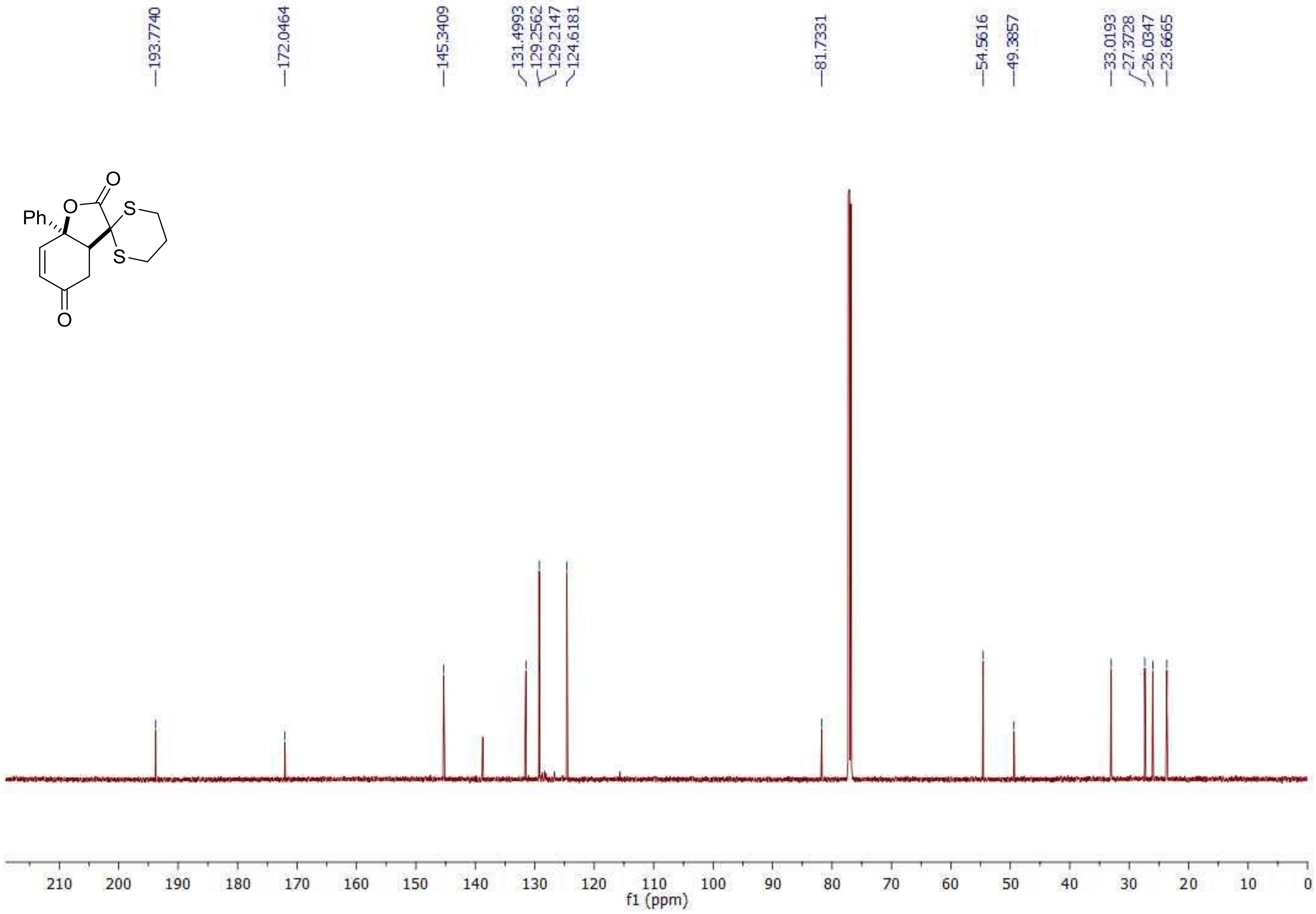
193.7740
172.0464
145.3409
131.4993
129.2562
129.2147
124.6181
81.7331
54.5616
49.3857
33.0193
27.3728
26.0347
23.6665
Ph
O
S
210 200 190 180 170 160 150 140 130 120 110 100 90 80 70 60 50 40 30 20 10 0
f1 (ppm)

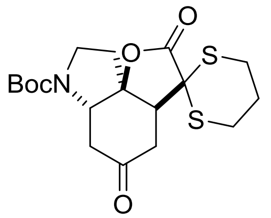
BocN
O
O
S
S
O

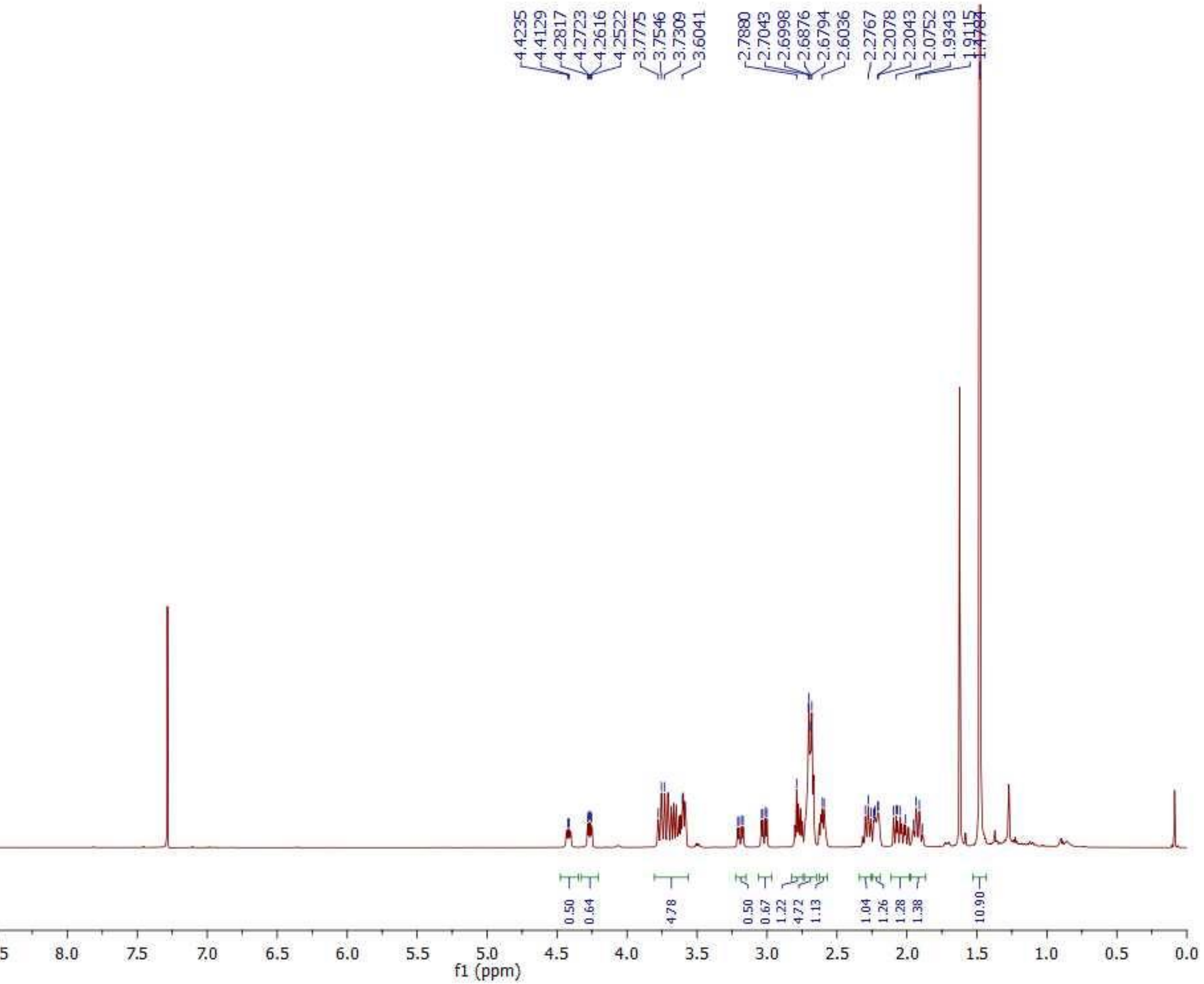
4.4235
4.4129
4.2817
4.2723
4.2616
4.2522
3.7775
3.7546
3.7309
3.6041
2.7880
2.7043
2.6998
2.6876
2.6794
2.6036
2.2767
2.2078
2.2043
2.0752
1.9343
1.9115
0.50
0.64
4.78
0.50
0.67
1.22
4.72
1.13
1.04
1.26
1.28
1.38
10.90
10.0
9.5
9.0
8.5
8.0
7.5
7.0
6.5
6.0
5.5
5.0
4.5
4.0
3.5
3.0
2.5
2.0
1.5
1.0
0.5
0.0
f1 (ppm)

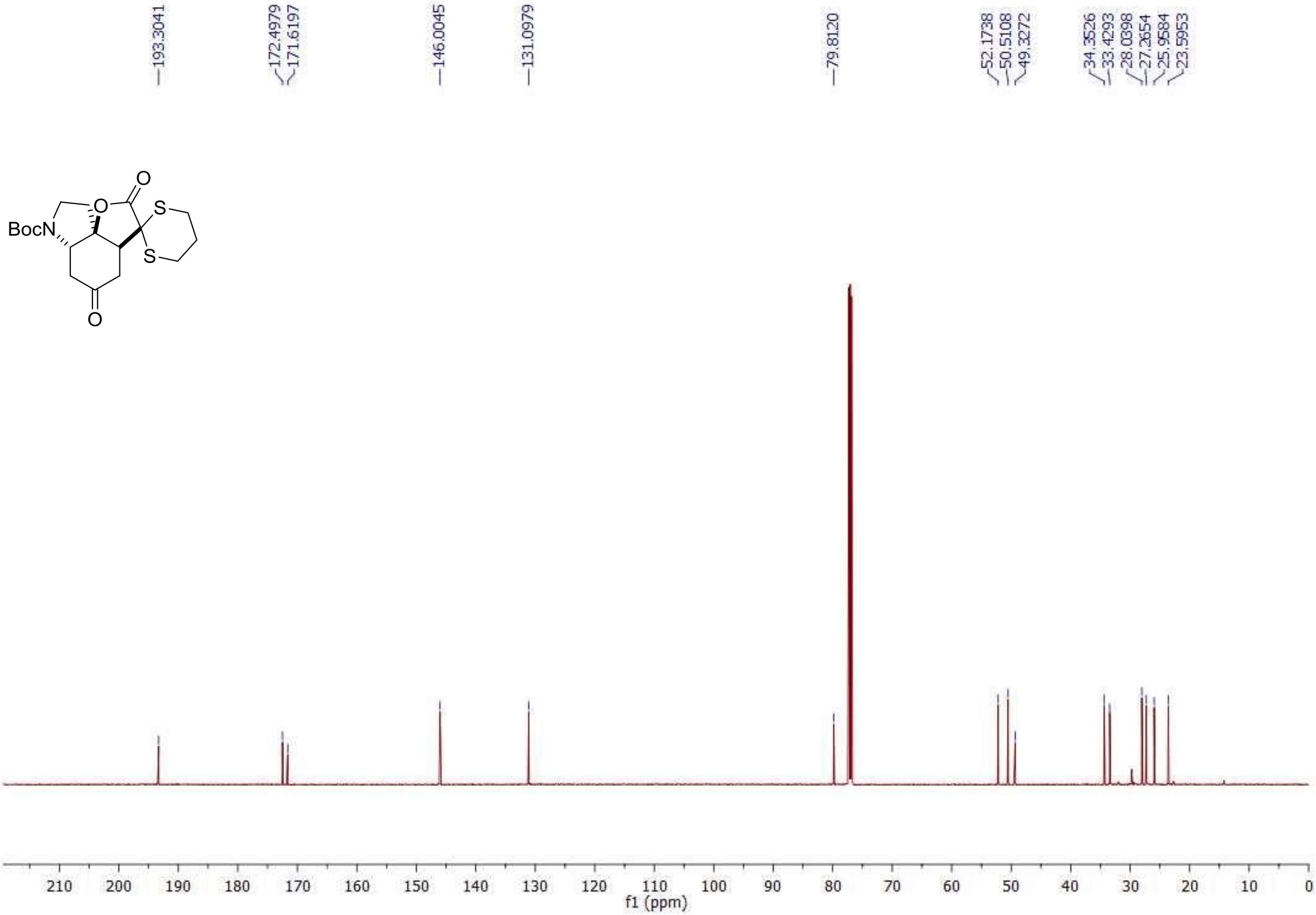
193.3041
172.4979
171.6197
146.0045
131.0979
79.8120
52.1738
50.5108
49.3272
34.3526
33.4293
28.0398
27.2654
25.9584
23.5953
BocN
O
O
S
S
O
210
200
190
180
170
160
150
140
130
120
110
100
90
80
70
60
50
40
30
20
10
0
f1 (ppm)

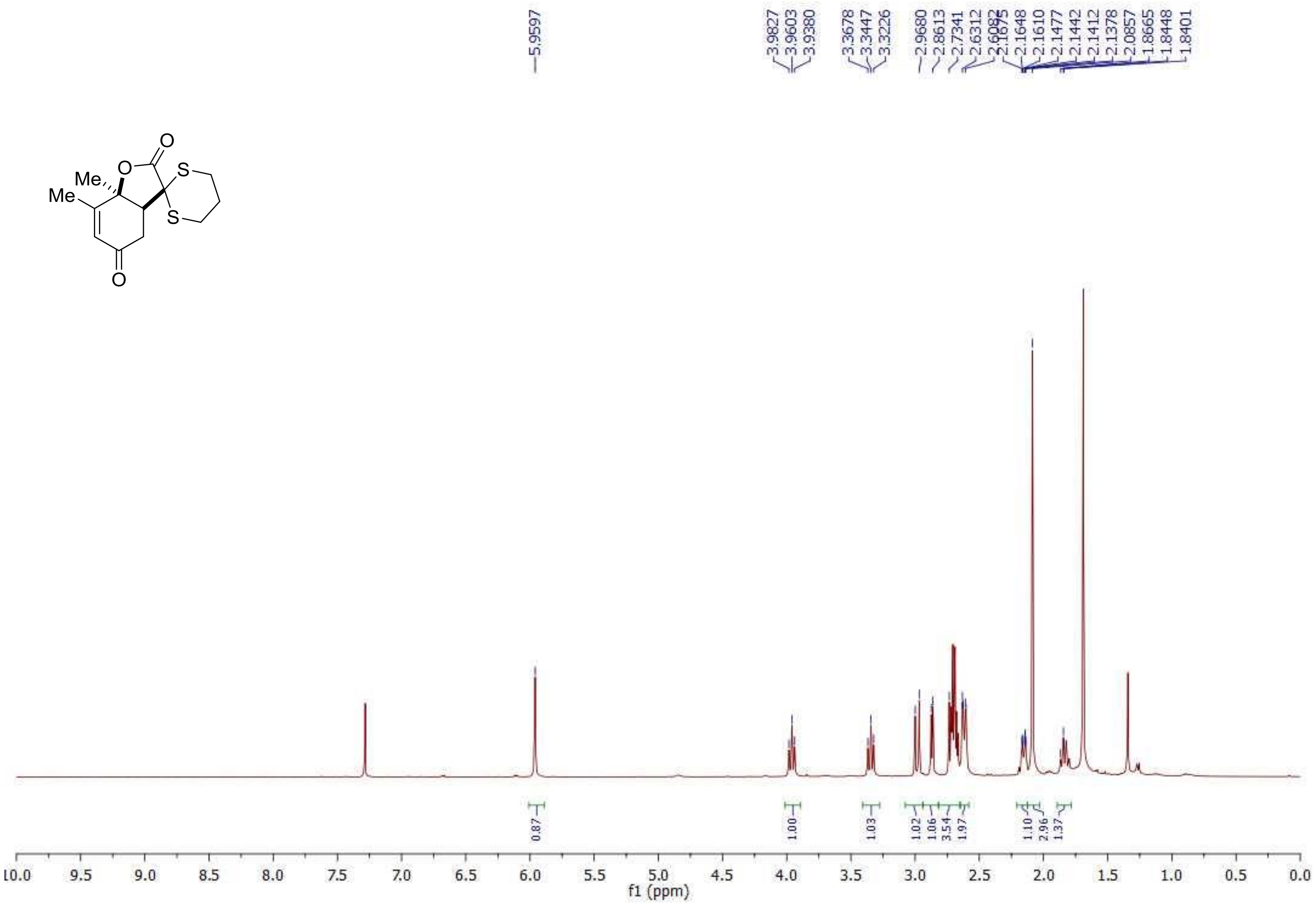

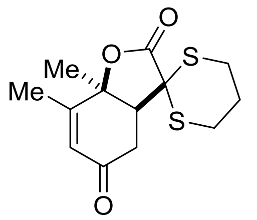

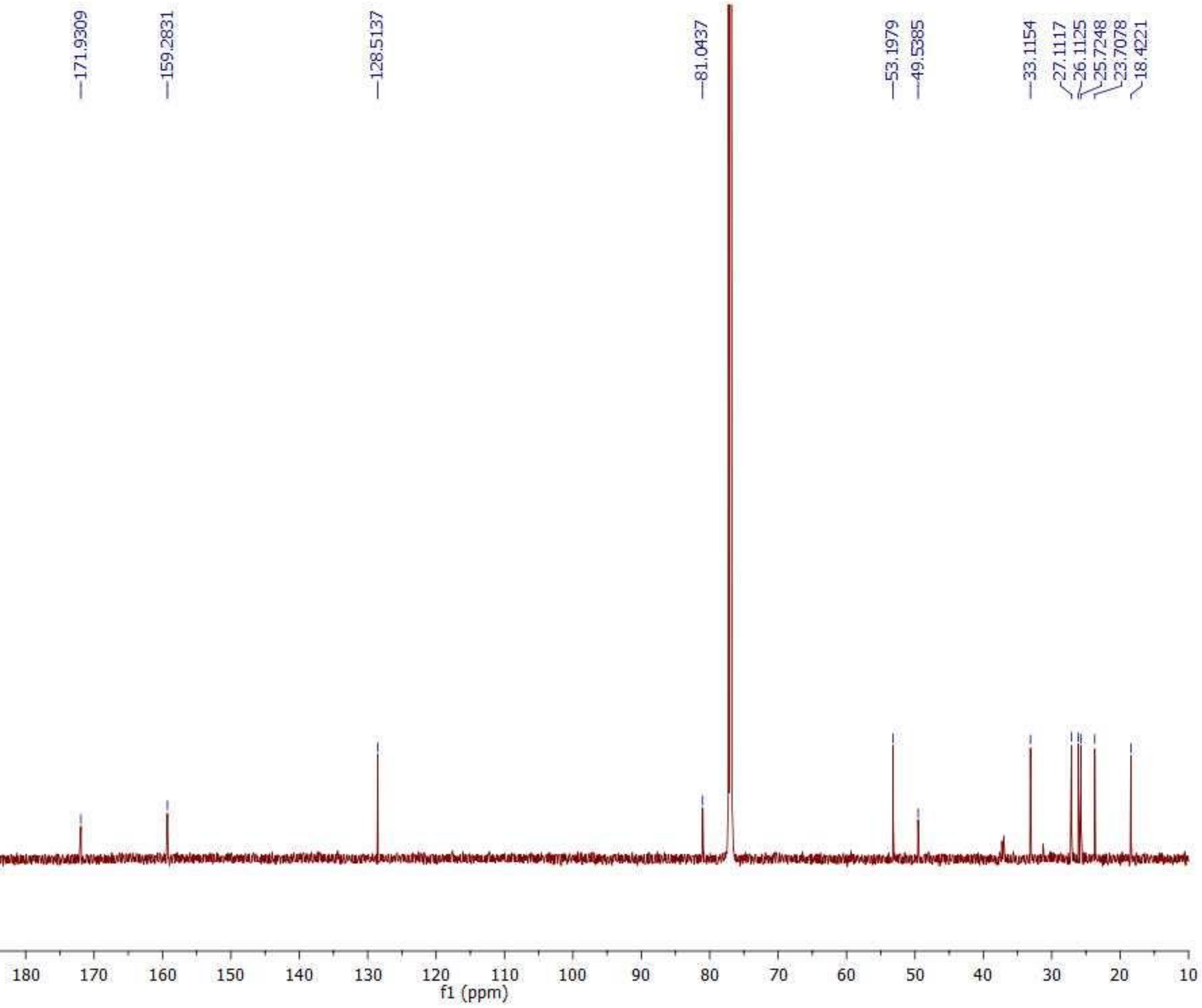

193.1661
171.9309
159.2831
128.5137
81.0437
53.1979
49.5385
33.1154
27.1117
26.1125
25.7248
23.7078
18.4221
210
200
190
180
170
160
150
140
130
120
110
100
90
80
70
60
50
40
30
20
10
f1 (ppm)

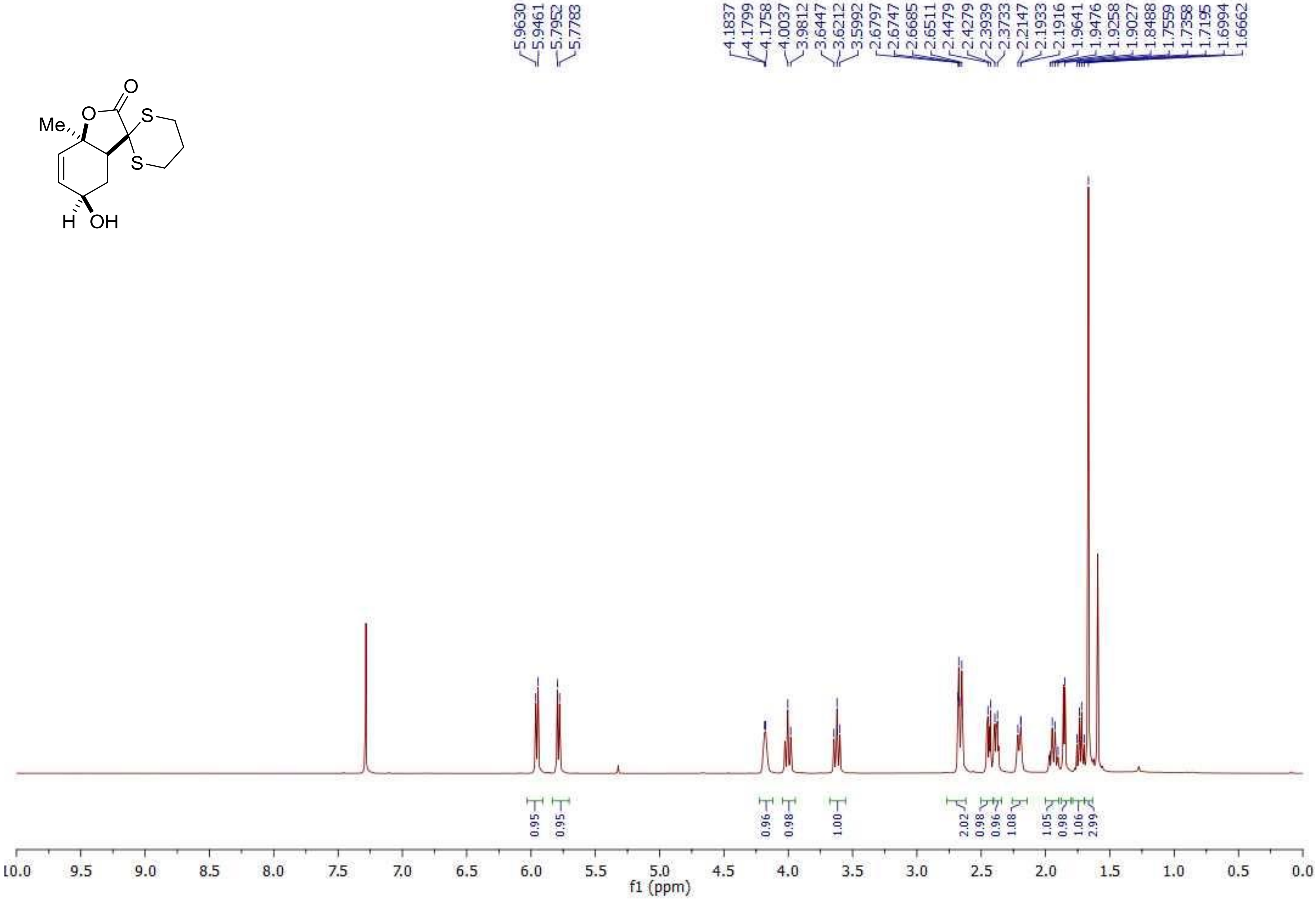

O
Me
O
S
S
H
OH
5.9630
5.9461
5.7952
5.7783
4.1837
4.1799
4.1758
4.0037
3.9812
3.6447
3.6212
3.5992
2.6797
2.6747
2.6685
2.6511
2.4479
2.4279
2.3939
2.3733
2.2147
2.1933
2.1916
1.9641
1.9476
1.9258
1.9027
1.8488
1.7559
1.7358
1.7195
1.6994
1.6662
0.95
0.95
0.96
0.98
1.00
2.02
0.98
0.96
1.08
1.05
0.98
1.06
2.99
10.0
9.5
9.0
8.5
8.0
7.5
7.0
6.5
6.0
5.5
5.0
4.5
4.0
3.5
3.0
2.5
2.0
1.5
1.0
0.5
0.0
f1 (ppm)

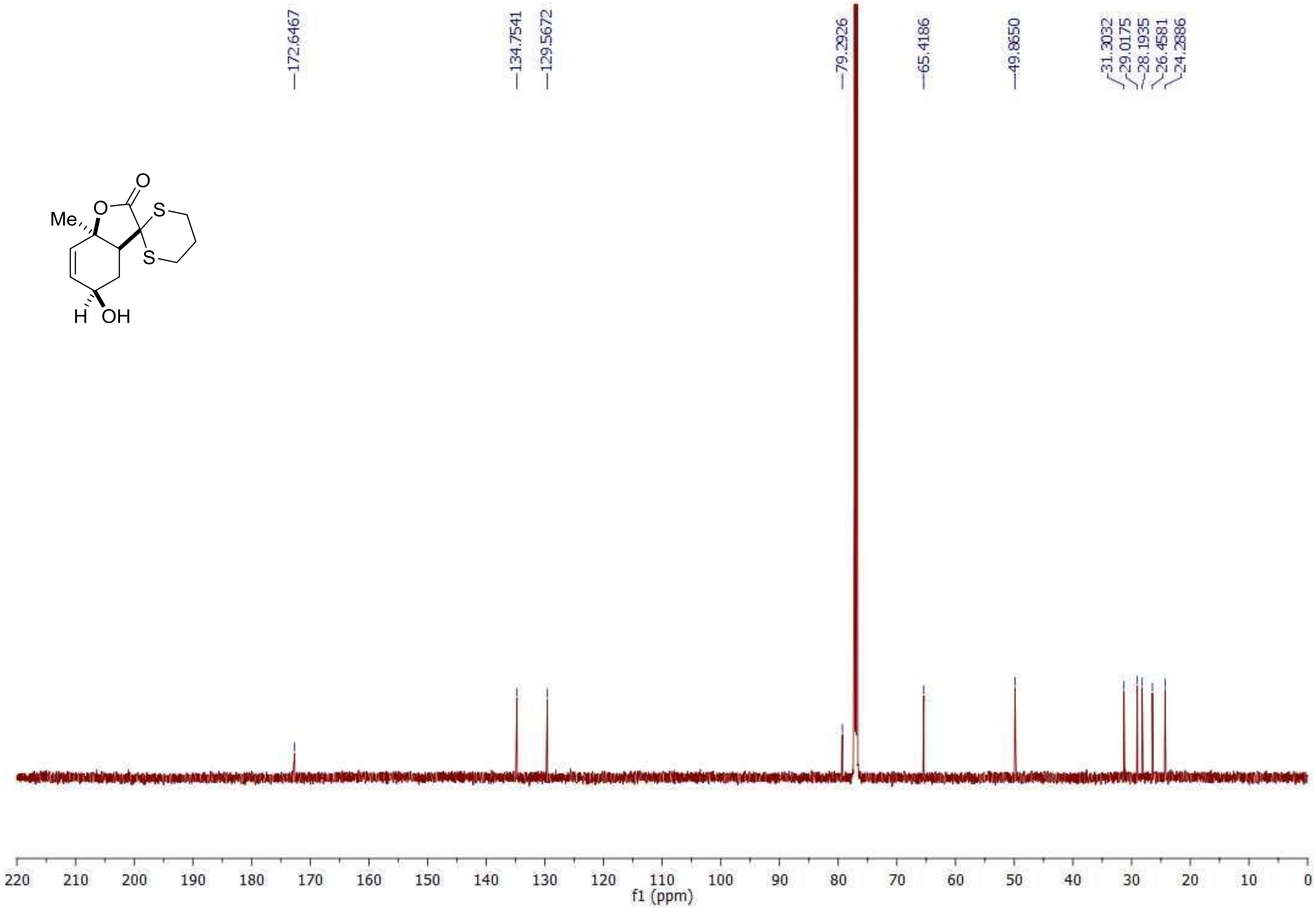
172.6467
134.7541
129.5672
79.2926
65.4186
49.8650
31.3032
29.0175
28.1935
26.4581
24.2886
220 210 200 190 180 170 160 150 140 130 120 110 100 90 80 70 60 50 40 30 20 10 0
f1 (ppm)

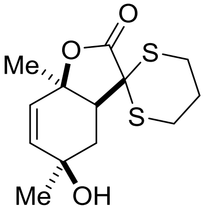
O
Me
O
S
S
Me
OH

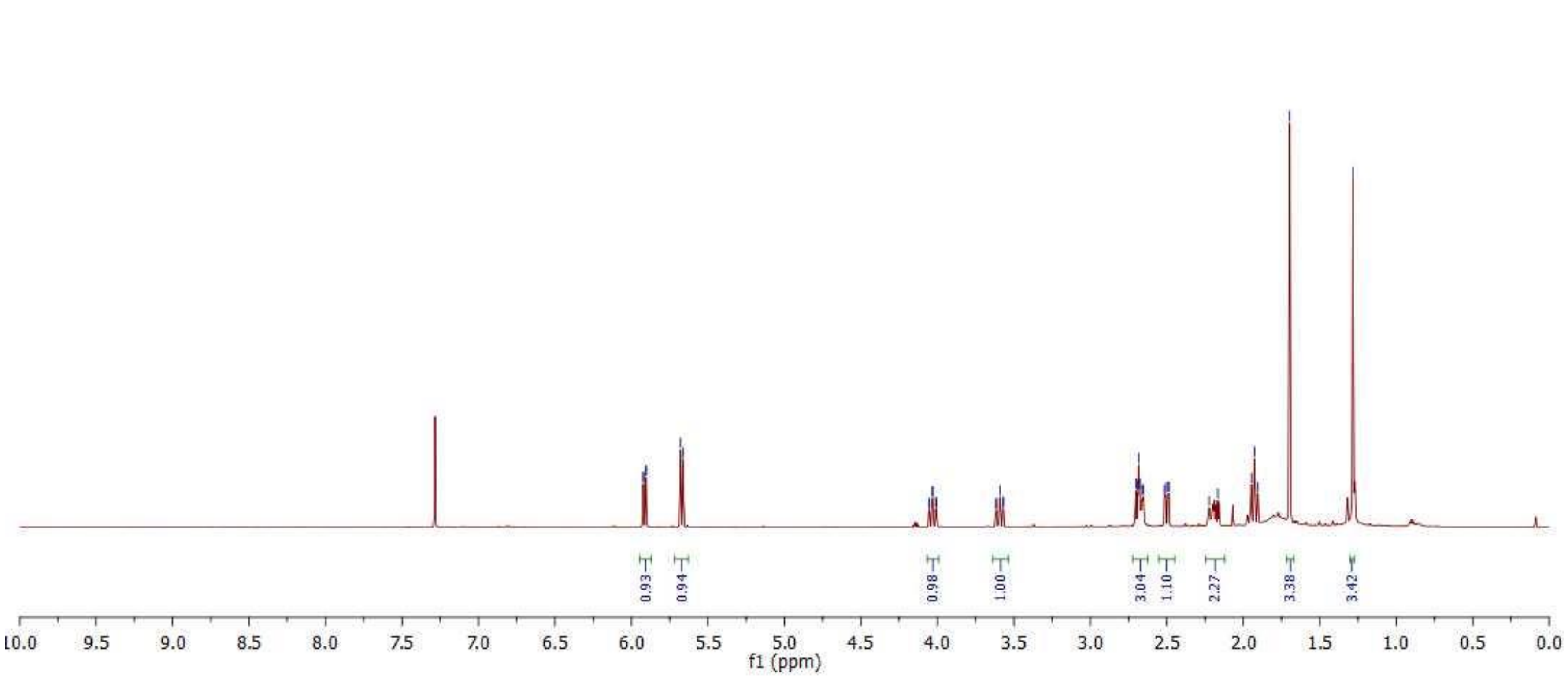
5.9224
5.9203
5.9055
5.9035
5.6788
5.6620
4.0560
4.0517
4.0330
4.0295
4.0109
4.0066
3.6128
3.5916
3.5712
3.5674
2.7007
2.6901
2.6838
2.6769
2.6537
2.5071
2.4954
2.4860
1.9469
1.9259
1.9047
1.2825
0.93
0.94
0.98
1.00
3.04
1.10
2.27
3.38
3.42
10.0
9.5
9.0
8.5
8.0
7.5
7.0
6.5
6.0
5.5
5.0
4.5
4.0
3.5
3.0
2.5
2.0
1.5
1.0
0.5
0.0
f1 (ppm)

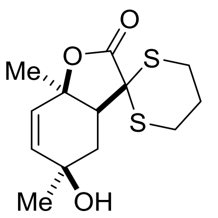

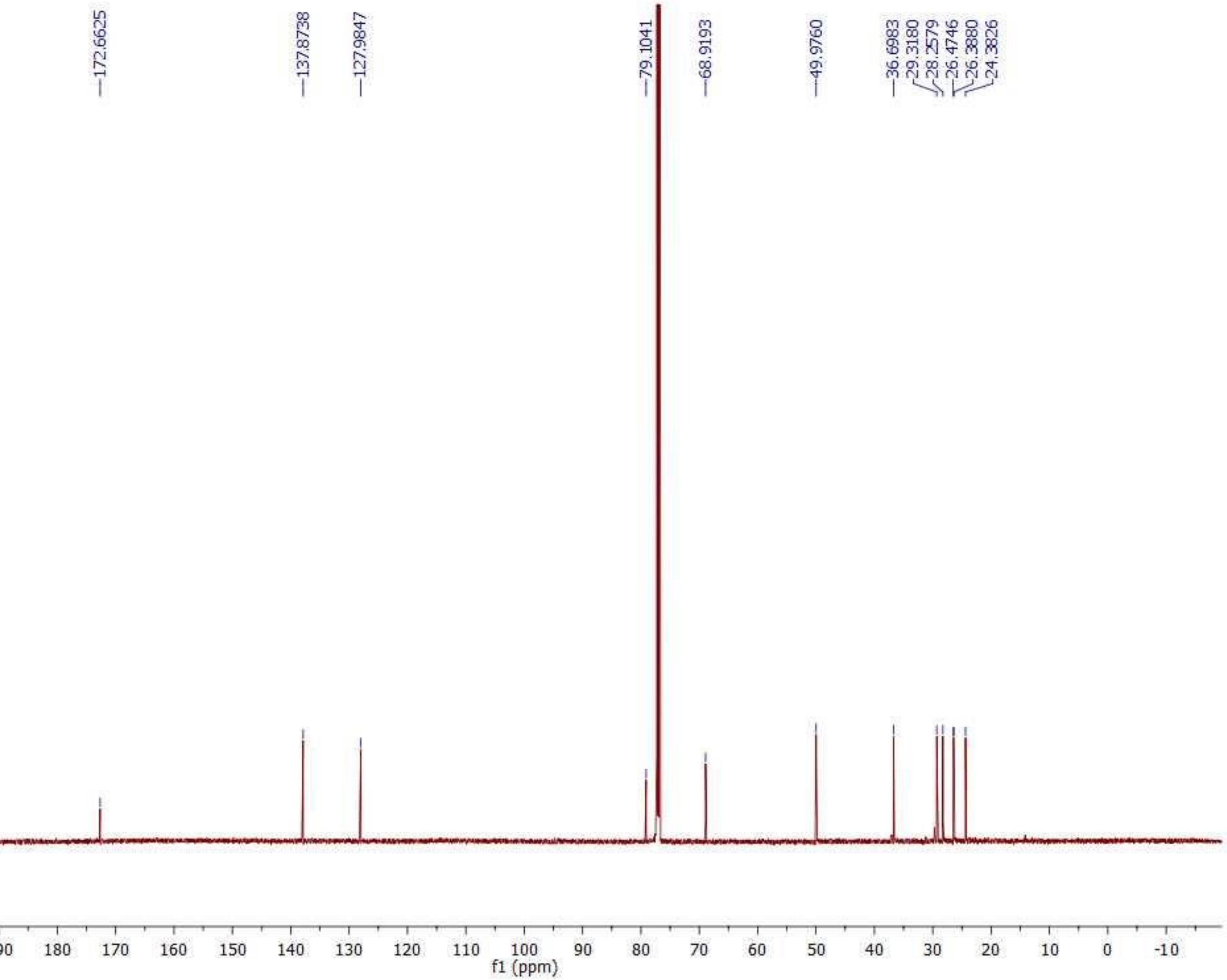
172.6625
137.8738
127.9847
79.1041
68.9193
49.9760
36.6983
29.3180
28.2579
26.4746
26.3880
24.3826
210 200 190 180 170 160 150 140 130 120 110 100 90 80 70 60 50 40 30 20 10 0 -10
f1 (ppm)
